# Maladaptive myelination promotes generalized epilepsy progression

**DOI:** 10.1038/s41593-022-01052-2

**Published:** 2022-05-02

**Authors:** Juliet K. Knowles, Haojun Xu, Caroline Soane, Ankita Batra, Tristan Saucedo, Eleanor Frost, Lydia T. Tam, Danielle Fraga, Lijun Ni, Katlin Villar, Sydney Talmi, John R. Huguenard, Michelle Monje

**Affiliations:** 1 Department of Neurology and Neurological Sciences, Stanford University, Stanford, CA USA; 2 Ph.D. Program in Human Biology, School of Integrative and Global Majors, University of Tsukuba, Tsukuba, Japan; 3 Howard Hughes Medical Institute, Stanford University, Stanford, CA USA

**Keywords:** Oligodendrocyte, Epilepsy

## Abstract

Activity-dependent myelination can fine-tune neural network dynamics. Conversely, aberrant neuronal activity, as occurs in disorders of recurrent seizures (epilepsy), could promote maladaptive myelination, contributing to pathogenesis. In this study, we tested the hypothesis that activity-dependent myelination resulting from absence seizures, which manifest as frequent behavioral arrests with generalized electroencephalography (EEG) spike-wave discharges, promote thalamocortical network hypersynchrony and contribute to epilepsy progression. We found increased oligodendrogenesis and myelination specifically within the seizure network in two models of generalized epilepsy with absence seizures (Wag/Rij rats and *Scn8a*^+/mut^ mice), evident only after epilepsy onset. Aberrant myelination was prevented by pharmacological seizure inhibition in Wag/Rij rats. Blocking activity-dependent myelination decreased seizure burden over time and reduced ictal synchrony as assessed by EEG coherence. These findings indicate that activity-dependent myelination driven by absence seizures contributes to epilepsy progression; maladaptive myelination may be pathogenic in some forms of epilepsy and other neurological diseases.

## Abstract (web summary)

Recurrent absence seizures aberrantly increase activity-regulated myelination within the seizure network; this maladaptive myelination, in turn, increases network hypersynchrony and seizure burden over time.

## Main

Neuronal activity can modulate myelin structure during development^1–3^ and throughout life^4–10^ by promoting proliferation of oligodendrocyte progenitor cells (OPCs), generation of new myelinating oligodendrocytes and changes to myelin structure^4–10^. Remodeling of myelin by existing oligodendrocytes can also occur in response to neuronal activity^9,10^. This plasticity of myelin and oligodendroglial cells has been best demonstrated to date in cortical and callosal axons^4–6,11^. Activity-regulated myelination is adaptive in the healthy brain, where it is hypothesized to increase neural network synchrony^7,12–14^, and myelin plasticity contributes to cognitive functions, including attention, learning and memory^7,11,14–17^.

The effects of myelin plasticity on network function in the healthy brain raise the question of how activity-regulated myelination may modulate network function in disease states characterized by abnormal patterns of neuronal activity, such as epilepsy. Diffusion-based imaging has demonstrated abnormal white matter microstructure in various forms of epilepsy in humans and rodent models^18–24^; however, definitive conclusions cannot be drawn about underlying myelin and axonal structure in the absence of gold-standard ultrastructural histology, nor is it known how altered white matter structure may contribute to epilepsy pathophysiology.

Absence seizures occur in multiple forms of human generalized epilepsy and are associated with behavioral arrest and generalized, but frontally predominant, spike-wave discharges^25,26^. Absence seizures originate in abnormal oscillations between the thalamus and cortex^27^ and propagate along myelinated tracts, including the anterior portions of the corpus callosum^28^. In humans and rodents, absence seizures are brief but very frequent, occurring hundreds of times per day^25,29^. Thus, generalized epilepsy with absence seizures presents an ideal paradigm to examine the relationship between activity-regulated myelination and seizure pathophysiology.

Genetic rodent models of generalized epilepsy with absence seizures exhibit defined periods of seizure onset followed by seizure progression, in which absence seizures increase in daily frequency over time^29,30^. This pattern of developmental seizure onset with progression is similar to the natural history of untreated, medically refractory and/or progressive forms of generalized epilepsy in children^31,25^. Blockade of seizures throughout the period of seizure progression in one model of absence epilepsy—Wag/Rij rats—prevents or delays seizure onset^29^, indicating that aberrant neuronal activity induces pathological network changes that contribute to subsequent progression in seizure burden. Although mechanisms of absence seizure onset and progression are incompletely understood, a well-documented feature is excessive synchrony (coordinated firing of groups of neurons) in the thalamocortical network^30,32^. Given the proposed effect of activity-regulated myelination on network synchrony^7,12–14^, we hypothesized that abnormally increased myelination within the seizure network, induced by absence seizures, might contribute to seizure progression.

## Results

### Increased seizure network myelination after epilepsy onset

**Fig. 1:**
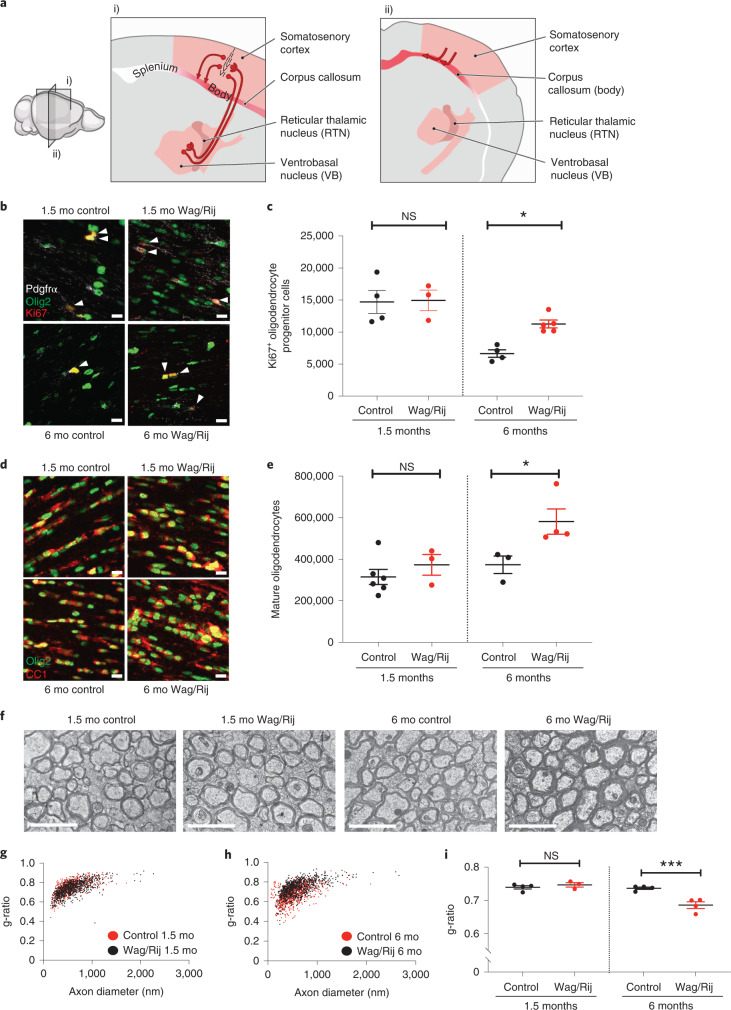
Increased oligodendrogenesis and myelination within the absence seizure network after epilepsy onset in Wag/Rij rats. **a**, Sagittal (**i**) and coronal (**ii**) views of the absence seizure network (pink/red). Absence seizures are maximal in ventrobasal and reticular thalamic nuclei and somatosensory cortices and propagate across the corpus callosum body, with little involvement of occipital cortices and corpus callosum splenium. Illustration by Sigrid Knemeyer at SciStories. **b**, Representative photomicrographs of dividing callosal OPCs expressing Olig2 (green), PDGFRα (white) and Ki67 (red), indicated with arrowheads. Scale bars, 10 μm. **c**, Total Ki67^+^ OPCs at 1.5 months and 6 months in control and Wag/Rij rats. 1.5 months, *n* = 4 control and 3 Wag/Rij rats; 6 months, *n* = 4 control and 5 Wag/Rij rats. One-way ANOVA: *F*_3,12_ = 10.43, *P* = 0.0012. Post hoc Sidak’s test comparing Ki67^+^ OPCs in Wag/Rij versus controls at 6 months: *P* = 0.025, and at 1.5 months: *P* = 0.99. **d**, Representative photomicrographs of callosal mature oligodendrocytes of 1.5-month-old and 6-month-old control or Wag/Rij rats, co-expressing Olig2 (green) and CC1 (red). Scale bars, 10 μm. **e**, Total callosal oligodendrocytes in control and Wag/Rij rats. 1.5 months, *n* = 6 control and 3 Wag/Rij rats; 6 months, *n* = 3 control and 4 Wag/Rij rats. One-way ANOVA: *F*_3,12_ = 6.558, *P* = 0.0071. Post hoc Sidak’s test comparing oligodendrocyte quantity in 6-month-old Wag/Rij rats versus controls: *P* = 0.029; 1.5-month-old: *P* = 0.65. **f**, Representative transmission electron micrographs of axon cross-sections in the mid-sagittal corpus callosum body. Scale bar, 2 μm. Scatter plots of individual axon g-ratios from 1.5-month-old (**g**) and 6-month-old (**h**) rats; each dot represents the g-ratio of one axon. **i**, Mean g-ratios for each rat; 1.5 months: *n* = 4 control and 3 Wag/Rij rats; 6 months: *n* = 4 control and 4 Wag/Rij rats. One-way ANOVA, *F*_3,11_ = 17.09, *P* = 0.0002. Post hoc Sidak’s test, control versus Wag/Rij at 1.5 months, *P* = 0.72, and 6 months, *P* = 0.0004. Each dot represents one rat (**c**, **e**, **i**); data are shown with group mean ± s.e.m. Black dots, control; red dots, Wag/Rij. *P < 0.05, **P < 0.01, ***P < 0.001, NS, non-significant (*P* > 0.05). Source data

To test the putative relationship between absence seizures and myelination, we used a well-established model of absence seizures—Wag/Rij rats^29^. Wag/Rij is an inbred rat strain that develops spontaneous, stereotyped absence seizures characterized by brief behavioral arrest, similar to absence seizures in humans^25^. The EEG correlate of these episodes in Wag/Rij rats is ~4–8-Hz, generalized, frontally predominant spike-wave discharges that are maximal over the somatosensory cortices^33^. Absence seizures arise from neurons connecting the thalamus and the cortex^27,34^. In rodents, absence seizures are particularly prominent in relays between the ventrobasal nuclear complex of the thalamus and somatosensory cortex, driven by complex circuitry involving interneurons of the reticular thalamic nucleus^27,30^. Seizures propagate throughout the brain via myelinated tracts, including the internal capsule (interconnects the thalamus and cortex) and the corpus callosum, a commissural tract that is required for seizure generalization^28^ (Fig. 1a). In Wag/Rij rats, infrequent seizures spontaneously begin around 2 months of age and steadily increase in daily frequency until the rate plateaus at 20–30 seizures per hour at about 6 months of age^29^. A closely related rat strain from which Wag/Rij is derived, Wistar, does not typically develop absence seizures during this time frame and, therefore, is used as a control for Wag/Rij rats^29^.

**Extended Data Fig. 1:**
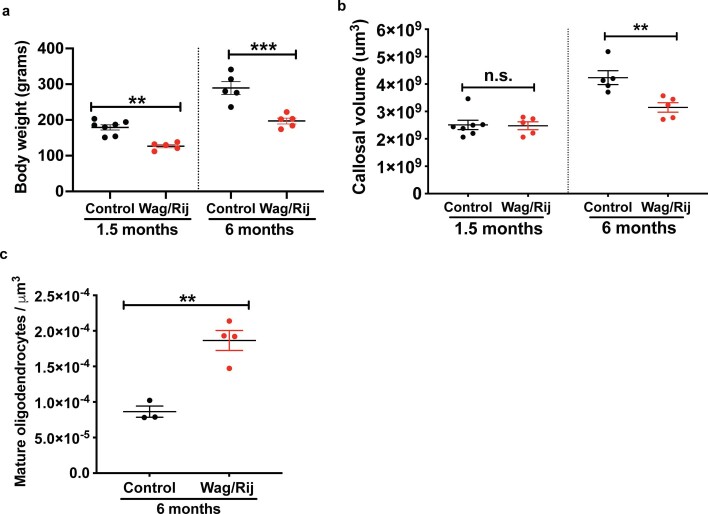
Callosal oligodendroglial cell density increases with absence seizures in Wag/Rij rats. **(a)** Control and Wag/Rij rat body weights. 1.5-month timepoint: control =7 rats; Wag/Rij =5 rats. 6-month timepoint: control=5 rats, Wag/Rij=5 rats. One-way ANOVA: F(3, 18)=39.83, p<0.0001. Sidak’s post-hoc testing: Wag/Rij vs. Wistar at 1.5-months, p=0.0032; at 6-months, p<0.0001. **(b)** Callosal volumes for each rat. 1.5-month timepoint: control=7 rats; Wag/Rij =5 rats. 6-month timepoint: control=5 rats, Wag/Rij=5 rats. One-way ANOVA: F(3, 18)=18.28, p<0.0001. Sidak’s post-hoc testing: Wag/Rij vs Wistar at 6-months: p=0.0022, at 1.5-months: p=0.9910. **(c)** Oligodendrocyte densities for each rat. Control=3 rats, Wag/Rij=4 rats. Two-tailed t-test, Wag/Rij vs control: p=0.0025. Each dot represents the mean for one rat, shown with group means ± SEM; control rats are represented with black dots and Wag/Rij rats are represented with red dots. *p<0.05, **p<0.01, ***p<0.001, n.s.=nonsignificant (p>0.05). Source data

**Extended Data Fig. 2:**
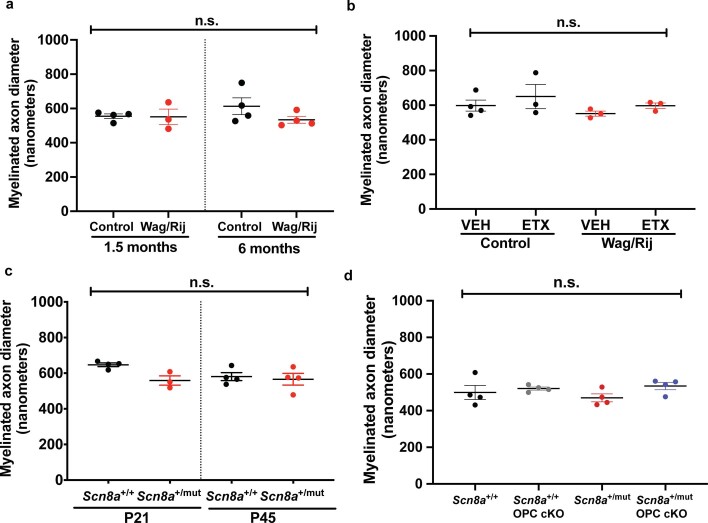
Myelinated axon diameters do not contribute to g-ratio differences in Wag/Rij rats and *Scn8a*^+/mut^ mice. **(a)** Mean diameters of myelinated axons for each rat. 1.5-month timepoint, n=4 control (black dots), 3 Wag/Rij (red dots); 6-month timepoint, n=4 control, 4 Wag/Rij rats. One-way ANOVA: F(3,11)=1.065, p=0.4033. **(b)** Myelinated axon diameter in the body of the corpus callosum at 7-months of age in control (black dots) and Wag/Rij (red dots) rats treated with vehicle (VEH) or ethosuximide (ETX). (Control-VEH, n=4 rats; Control-ETX, n=3 rats; Wag/Rij-VEH, n=3 rats; Wag/Rij-ETX, n=3 rats). One-way ANOVA: F(3,9)=0.9890, p=0.44. **(c)** Mean myelinated axon diameters for *Scn8a*^+/+^ (black dots) and *Scn8a*^+/mut^ (red dots) mice. P21, *Scn8a*^+/+^ n=4 mice; *Scn8a*^+/mut^ n=3 mice. P45, *Scn8a*^+/+^ n=4 mice; *Scn8a*^+/mut^ n=4 mice. One-way ANOVA: F(3,11)=2.822, p=0.088. **(d)** Myelinated axon diameters in the body of the corpus callosum in 6-month-old *Scn8a*^+/+^ (black dots, n=4 mice), *Scn8a*^+/+^ OPC cKO (gray dots, n=4 mice), *Scn8a*^+/mut^ (red dots, n=4 mice) and *Scn8a*^+/mut^ OPC cKO mice (blue dots, n =4 mice). One-way ANOVA: F(3,12) =1.324, p=0.31. Each dot represents the mean for one rat or mouse, shown with group means ± SEM. *p<0.05, **p<0.01, ***p<0.001, n.s.=nonsignificant (p>0.05). Source data

**Extended Data Fig. 3:**
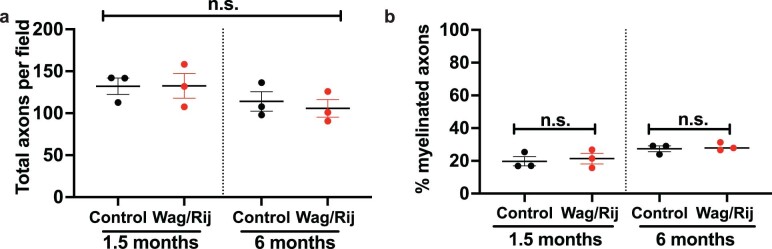
Percent axonal myelination and total axon number are similar in control and Wag/Rij rats. **(a)** Total number of axons per electron micrograph (field), for each rat (1.5-month timepoint: control=3 rats; Wag/Rij=3 rats. 6-month timepoint: control=3 rats, Wag/Rij=3 rats). One-way ANOVA: F(3, 8)=1.302, p=0.3388. **(b)** Percent of total callosal axons (myelinated + unmyelinated) that are myelinated in control and Wag/Rij rats. 1.5-month timepoint: control=3 rats; Wag/Rij=3 rats. 6-month timepoint: control=3 rats, Wag/Rij=3 rats. One-way ANOVA: F(3, 8)=3.337, p=0.0768. Sidak’s post-hoc test, percent myelination in Wag/Rij vs control rats at 1.5-months (p=0.8764), and at 6-months (p=0.9283). Each dot represents the mean for one rat, shown with group means ± SEM; control rats are represented with black dots and Wag/Rij rats are represented with red dots. *p<0.05, **p<0.01, ***p<0.001, n.s.=nonsignificant (p>0.05). Source data

**Extended Data Fig. 4:**
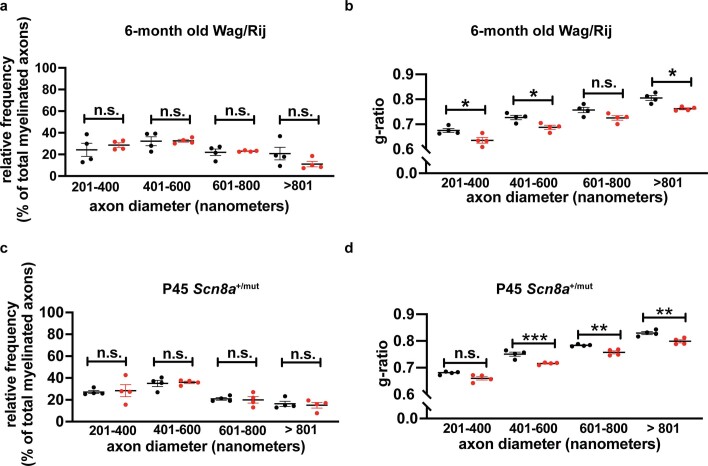
Increased myelin sheath thickness across axon diameters in Wag/Rij rats and *Scn8a*^+/mut^ mice. **(a)** Relative frequency of myelinated axons across axon diameters in 6-month old Wag/Rij (red dots) and control rats (black dots). N=4 control rats and 4 Wag/Rij rats. One-way ANOVA: F(7,24)=3.566, p = 0.009. Post-hoc Sidak’s testing, control vs. Wag/Rij: 201-400 nm, p=0.88; 401-600 nm, p=0.99; 601-800 nm, p=0.99; >801 nm p=0.27. **(b)** *g*-ratios in axon populations sorted by diameter. N=4 control rats and 4 Wag/Rij rats. One-way ANOVA: F(7,24)=36.79, p<0.0001. Sidak’s testing, control vs. Wag/Rij: 201-400 nm, p=0.0186; 401-600 nm, p=0.0194; 601-800 nm, p=0.0759; >801 nm p=0.0095. **(c)** Relative frequency of myelinated axons across axon diameters in P45 *Scn8a*^+/+^ mice (black dots) compared to *Scn8a*^+/mut^ (red dots). N=4 *Scn8a*^+/+^ and 4 *Scn8a*^+/mut^ mice. One-way ANOVA: F(7,24)=8.067, p<0.0001. Post-hoc analysis with Sidak’s testing, *Scn8a*^+/+^ vs. *Scn8a*^+/mut^ mice: 201-400 nm, p=0.9971; 401-600 nm, p=0.9988; 601-800 nm, p=0.9990; >801 nm p=0.9954. **(d)** *g*-ratios from *Scn8a*^+/+^ and *Scn8a*^+/mut^ mice, sorted by axon diameter (N=4 *Scn8a*^+/+^ and 4 *Scn8a*^+/mut^ mice). One-way ANOVA: F(7,24)=126.2, p<0.0001. Sidak’s test, *Scn8a*^+/+^ vs. *Scn8a*^+/mut^ *g*-ratios: 201-400 nm, p=0.0509; 401-600 nm, p=0.0003; 601-800 nm, p=0.0059; >801 nm p=0.0013. Each dot represents the mean for one rat or mouse, shown with group means ± SEM. *p<0.05, **p<0.01, ***p<0.001, n.s.=nonsignificant (p>0.05). Source data

To investigate whether absence seizures cause aberrant activity-regulated myelination within the seizure network, we began by assessing the proliferation of OPCs together with the number of mature oligodendrocytes in the mid-region (body) of the corpus callosum, focusing specifically on the area interconnecting the somatosensory cortices that is involved in the absence seizure network (Allen Brain Atlas, http://atlas.brain-map.org/). Given anatomical differences between Wag/Rij and Wistar (control) rats (Extended Data Fig. 1a), we used unbiased stereological methods to assess total cell numbers (Fig. 1b–e) as well as volume of the corpus callosum (Extended Data Fig. 1b) and oligodendrocyte density (Extended Data Fig. 1c). Before seizure onset, at 1.5 months of age, control rats and Wag/Rij (seizure) rats exhibit equivalent OPC proliferation in the corpus callosum. However, at 6 months of age, when seizures are well-established, Wag/Rij rats exhibit a 69% increase in dividing OPCs relative to age-matched control rats (cells co-expressing Ki67, PDGFRα and Olig2; 6,647 ± 585 dividing OPCs in control versus 11,259 ± 615 dividing OPCs in Wag/Rij; Fig. 1b–c). Compared to age-matched control rats, Wag/Rij rats also exhibit a 56% increase in Olig2, CC1-expressing mature oligodendrocytes at 6 months of age, indicative of increased oligodendrogenesis (mean ± s.e.m.: 373,697 ± 42,161 mature oligodendrocytes in control versus 581,490 ± 60,898 in Wag/Rij; Fig. 1d–e and Extended Data Fig. 1c). Both the total number and density of oligodendrocytes were increased, indicating that these differences were not a passive consequence of differences in callosal volume. In contrast, Wag/Rij and control rats exhibit similar numbers of oligodendrocytes at 1.5 months of age, before seizure onset (Fig. 1d–e). Given these findings indicating that oligodendrogenesis increases in parallel with seizure progression, we next investigated whether myelin structure is also altered. We used transmission electron microscopy to visualize cross-sections of myelinated axons in the mid-sagittal plane of the body of the corpus callosum (Fig. 1f), where oligodendrogenesis was assessed. We measured myelin sheath thickness relative to axon diameter, g-ratio^4,6,11,35^, in 1.5-month-old and 6-month-old Wag/Rij rats and Wistar controls. We found an increase in mean myelin sheath thickness (decreased g-ratio) in 6-month-old Wag/Rij rats compared to controls (control: 0.7368 ± 0.0036, Wag/Rij: 0.6862 ± 0.0099; Fig. 1h,i). This difference in myelin sheath thickness is similar in magnitude to functionally relevant changes in previous studies^4,6,11^ and is meaningful particularly considering that the dynamic range of central nervous system g-ratios is typically ~0.6–0.8^4,6,11,35^. Differences in g-ratios were not observed before seizure onset at 1.5 months (Fig. 1g,i) and are not attributable to strain differences in axon diameter or total axon number either before (1.5 months) or after (6 months) onset of seizures (Extended Data Figs. 2a and 3a). There was no difference in the percentage of axons that were myelinated in Wag/Rij and controls rats at either 1.5 months or 6 months (Extended Data Fig. 3b); changes in myelin sheath thickness observed at 6 months were not restricted to a particular range of axon diameters (Extended Data Fig. 4a,b).

**Extended Data Fig. 5:**
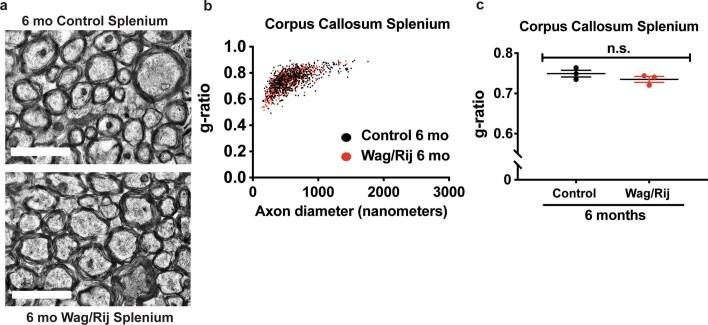
Increased myelination is specific to the absence seizure network. **(a)** Representative transmission electron micrographs showing similar appearing myelinated axons in the splenium of a 6-month-old control rat (upper panel) and a Wag/Rij rat (lower panel). Scale bar=2 μm. **(b)** Scatterplot of *g*-ratios showing overlap between control and Wag/Rij rats’ *g*-ratios. Each data point represents one axon’s *g*-ratio, with control *g*-ratios in black and Wag/Rij *g*-ratios in red. n=3 control, 3 Wag/Rij rats. **(c)** Each dot represents the mean *g*-ratio for one rat. n=3 control, 3 Wag/Rij rats. Data are shown with group means ± SEM and were analyzed with a two-tailed t-test (n.s., non-significant, p=0.27). Source data

Absence seizures in rodents are most prominent in the somatosensory cortices^33^. We reasoned that, if abnormally increased myelination is caused by seizure activity, these changes would be specific to the seizure-affected regions. Therefore, we assessed myelin in the posterior corpus callosum (splenium), connecting cortical regions where seizure activity is less prominent in humans and rodents^26,33^. The seizure-associated myelin difference observed in the body of the corpus callosum was not found in the splenium, as shown in Extended Data Fig. 5. Taken together, these data demonstrate increased oligodendrogenesis and abnormally increased myelination in a temporal and anatomical pattern that parallels seizure activity.

### Seizures are necessary for aberrant callosal myelination

**Fig. 2:**
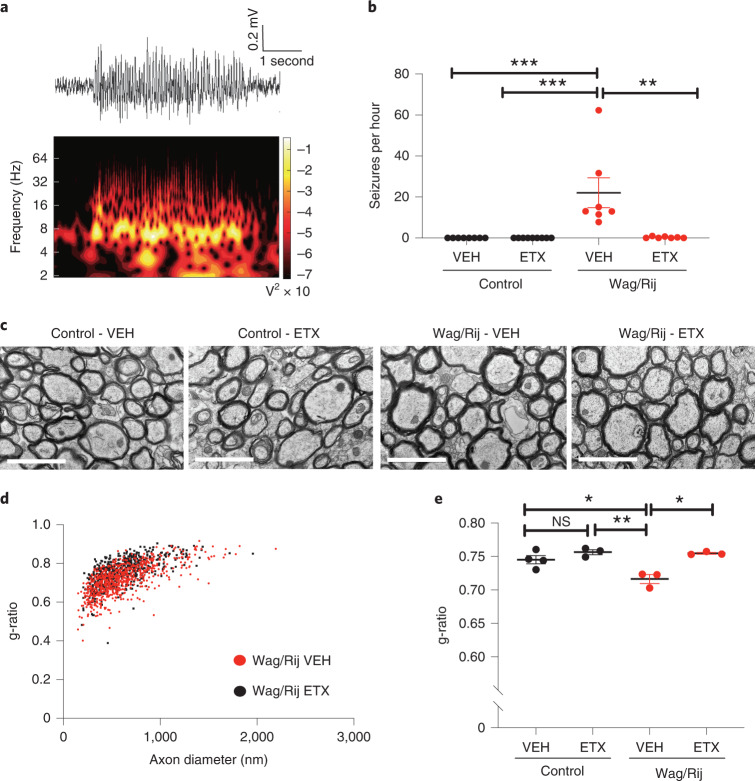
Seizures are necessary for aberrant callosal myelination. **a**, Representative spike-wave discharge seizure from a 6.5-month-old VEH-treated Wag/Rij rat (upper panel); spectral analysis demonstrating that the predominant seizure frequency is ~8 Hz (lower panel). **b**, Mean seizures per hour for each rat. Control-VEH, *n* = 8 rats; Control-ETX, *n* = 9 rats; Wag/Rij-VEH, *n* = 7 rats; Wag/Rij-ETX, *n* = 7 rats. Kruskal–Wallis analysis revealed significant variance in seizure burden (seizures per hour) between groups (Kruskal–Wallis statistic, 25.14, *P* < 0.0001). Dunn’s post hoc testing: Control-VEH versus Wag/Rij-VEH, *P* < 0.0001, Control-ETX versus Wag/Rij-VEH, *P* < 0.0001, Wag/Rij-VEH versus Wag/Rij-ETX, *P* = 0.0099. **c**, Representative transmission electron micrographs from the mid-sagittal body of the corpus callosum of 7-month-old rats. Scale bars, 2 μm. **d**, Scatter plots of g-ratios in 7-month-old VEH-treated or ETX-treated Wag/Rij rats. Each dot represents the g-ratio of one axon. **e**, Mean g-ratios for each 7-month-old Wag/Rij rat and control rat from measurements shown in **d**. Control-VEH, *n* = 4 rats; Control-ETX, *n* = 3 rats; Wag/Rij-VEH, *n* = 3 rats; Wag/Rij-ETX, *n* = 3 rats. One-way ANOVA revealed significant variance in group g-ratios *F*_3,9_ = 11.36, *P* = 0.0021. Tukey testing with corrections for multiple comparisons revealed decreased g-ratio (increased myelin thickness) in Wag/Rij-VEH rats with seizures compared to control rats (Control-VEH versus Wag/Rij-VEH, *P* = 0.015, and Control-ETX versus Wag/Rij-VEH, *P* = 0.0028). This increase in myelin sheath thickness was prevented with seizure blockade by ETX (Wag/Rij-VEH versus Wag/Rij-ETX, *P* = 0.0038), which normalized g-ratios (Control-VEH versus Wag/Rij-ETX, *P* = 0.5841, and Control-ETX versus Wag/Rij-ETX, *P* = 0.9952). ETX treatment did not alter g-ratios in control rats (Control-VEH versus Control-ETX, *P* = 0.4492). Each dot represents the mean for one rat (**b**, **e**) shown with group means ± s.e.m.; control rats are represented with black dots, and Wag/Rij rats are represented with red dots. *P < 0.05, **P < 0.01, ***P < 0.001, NS, non-significant (*P* > 0.05). VEH, vehicle; ETX, ethosuximide. Source data

We reasoned that absence seizures likely induce aberrant activity-regulated myelination. To confirm that seizures are required for the observed increase in callosal myelination, we treated Wag/Rij and control rats with the anti-seizure drug ethosuximide (ETX) at ~300 mg/kg/day, a dose known to prevent or reduce seizures in Wag/Rij rats^29^. This dosing led to a mean plasma concentration of 101.3 ± 10.33 µg ml^−1^ (mean ± s.e.m., *n* = 20 rats), without signs of toxicity and similar to therapeutic levels in humans, typically between 40 µg ml^−1^ and 100 µg ml^−1^ (https://pubchem.ncbi.nlm.nih.gov/compound/Ethosuximide). Treatment was initiated at 1.5 months of age, before seizure onset. After 5 months of treatment, EEG at 6.5 months of age revealed frequent absence seizures of 5 +/- 0.5 second duration in vehicle-treated Wag/Rij rats (Fig. 2a,b) as previously described^29^. ETX robustly decreased or prevented seizures, as expected (Fig. 2b). We examined callosal myelination in control and Wag/Rij rats after vehicle or ETX administration at 7 months of age. Similarly to the findings described in Fig. 1, myelin sheath thickness was increased in vehicle-treated 7-month-old Wag/Rij rats compared to controls. However, seizure blockade with ETX treatment normalized myelin sheath thickness (g*-*ratio) in Wag/Rij rats (Fig. 2c–e). ETX did not influence g-ratio in control rats (Fig. 2e), nor did it affect axonal diameter in any group (Extended Data Fig. 2b).

Together, these findings indicate that absence seizures increase myelination specifically within the seizure-affected network and suggest a mechanism of aberrantly increased activity-dependent myelination that could be deleterious (maladaptive), contributing to epilepsy pathogenesis. To further test this hypothesis, we sought to evaluate seizure-related myelin changes in a second model of absence seizures.

### Increased myelination in a second model of generalized epilepsy

We next quantified oligodendrogenesis and myelin structure in a second, distinct rodent model of generalized epilepsy with absence seizures—*Scn8a*^+/mut^ mice. *Scn8a*^+/mut^ mice bear a heterozygous loss-of-function mutation in the voltage-gated sodium channel *Nav1.6*, which results in interneuron dysfunction in the reticular thalamic nucleus, thalamocortical hyper-synchrony and spontaneous 4–8-Hz absence seizures^30,36^. *Scn8a*^+/mut^ mice exhibit seizures that begin around post-natal day (P) 21 and steadily increase in number until P35–P45^30^. The use of this mouse model confers the advantage of wild-type littermates on a congenic background and the opportunity for targeted genetic manipulation of activity-dependent myelination.

**Fig. 3:**
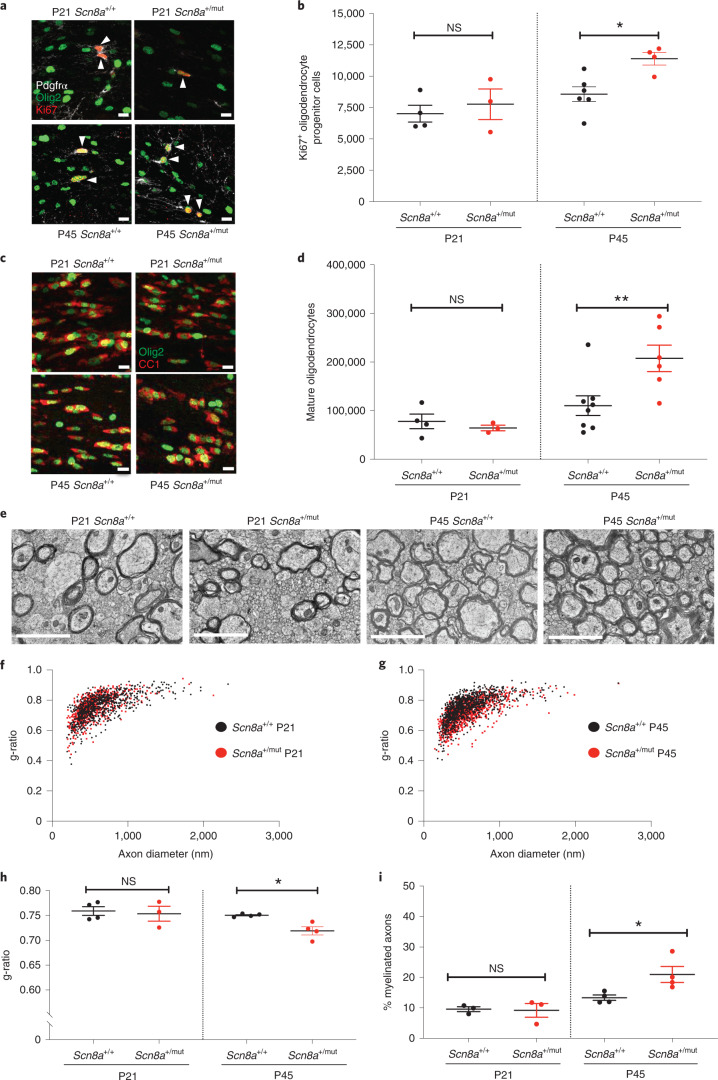
Increased oligodendrogenesis and myelination in *Scn8a*^*+/mut*^ mice after epilepsy onset. **a**, Representative photomicrographs of dividing callosal OPCs (Ki67: red, PDGFRα: white, Olig2: green), indicated with arrowheads. Scale bars, 10 μm. **b**, Total callosal Ki67^+^ OPCs in *Scn8a*^+/+^ and *Scn8a*^+/mut^ mice. P21, *Scn8a*^+/+^ *n* = 4 mice; *Scn8a*^+/mut^ *n* = 3. P45, *Scn8a*^+/+^ *n* = 6 mice; *Scn8a*^+/mut^ *n* = 4. One-way ANOVA: *F*_3,13_ = 6.684; *P* = 0.0057. Post hoc Sidak’s test, Ki67^+^ OPCs in *Scn8a*^+/mut^ versus *Scn8a*^+/+^ at P45: *P* = 0.021; at P21: *P* = 0.76. **c**, Representative photomicrographs of callosal mature oligodendrocytes expressing CC1 (red) and Olig2 (green). Scale bars, 10 μm. **d**, Total callosal oligodendrocytes in *Scn8a*^+/+^ and *Scn8a*^+/mut^ mice. P21, *Scn8a*^+/+^ *n* = 4 mice; *Scn8a*^+/mut^ *n* = 3 mice. P45, *Scn8a*^+/+^ *n* = 8 mice; *Scn8a*^+/mut^ *n* = 6 mice. One-way ANOVA: *F*_3,17_ = 7.382; *P* = 0.0022. Post hoc Sidak’s test, *Scn8a*^+/mut^ versus *Scn8a*^+/+^ mice at P45: *P* = 0.0069; at P21, *P* = 0.94. **e**, Representative transmission electron micrographs of callosal axons in P21 and P45 *Scn8a*^+/+^ and *Scn8a*^+/mut^ mice. Scale bar, 2 μm. **f**, **g**, Scatter plots of individual axon g-ratios from P21 (**f**) and P45 (**g**) mice. Each dot represents the g-ratio of one axon. **h**, Mean g-ratios from *Scn8a*^+/+^ and *Scn8a*^+/mut^ mice. P21, *Scn8a*^+/+^ *n* = 4 mice; *Scn8a*^+/mut^ *n* = 3 mice. P45, *Scn8a*^+/+^ *n* = 4 mice; *Scn8a*^+/mut^ *n* = 4 mice. One-way ANOVA: *F*_3,11_ = 4.471; *P* = 0.028. Post hoc Sidak’s test: g-ratios in *Scn8a*^+/mut^ versus *Scn8a*^+/+^ at P45, *P* = 0.046; at P21, *P* = 0.89. **i**, Percentage of total callosal axons that are myelinated in *Scn8a*^+/+^ and *Scn8a*^+/mut^ mice. P21: *Scn8a*^+/+^ *n* = 3; *Scn8a*^+/mut^ *n* = 3. P45: *Scn8a*^+/+^ *n* = 4, *Scn8a*^+/mut^ *n* = 4. One-way ANOVA: *F*_3,10_ = 8.565, *P* = 0.0041. Post hoc Sidak’s test: percent myelination in *Scn8a*^+/+^ versus *Scn8a*^+/mut^ at P21, *P* = 0.9897; at P45, *P* = 0.0248. Each dot represents the mean for one mouse (**b**, **d**, **h**, **i**) shown with group means ± s.e.m.; *Scn8a*^+/+^ mice are represented by black dots, and *Scn8a*^+/mut^ mice are represented with red dots. *P < 0.05, **P < 0.01, ***P < 0.001, NS, non-significant (*P* > 0.05). Source data

**Extended Data Fig. 6:**
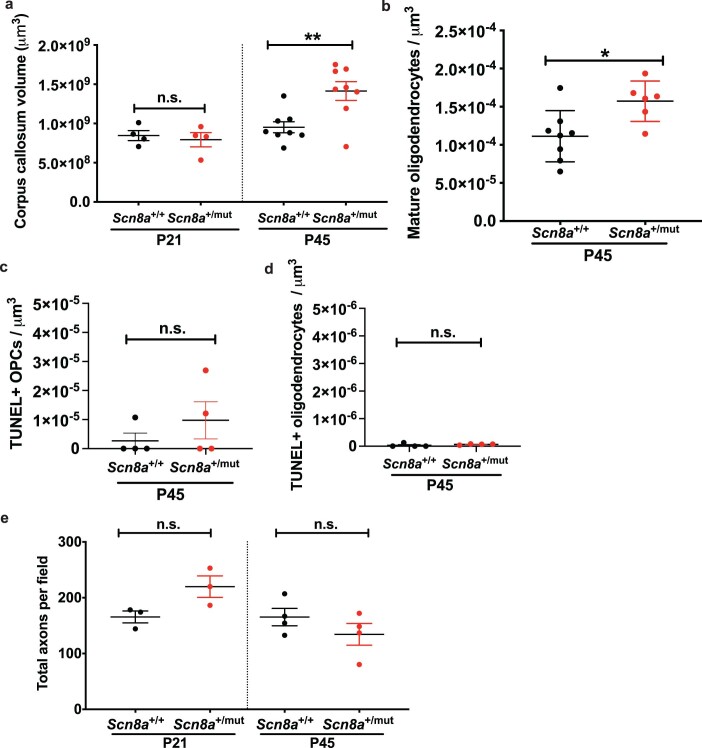
Increased callosal oligodendroglial cell density and equivalent callosal axon density in *Scn8*a^+/mut^ mice. **(a)** Callosal volumes for *Scn8a*^+/+^ and *Scn8a*^+/mut^ mice. P21: *Scn8a*^+/+^=4 mice; *Scn8a*^+/mut^=4 mice. P45: *Scn8a*^+/+^=8 mice, *Scn8a*^+/mut^=8 mice. One-way ANOVA: F(3,20)=8.443; p=0.0008. Sidak’s post-hoc test, *Scn8a*^+/mut^ vs *Scn8a*^+/+^ at P45, p=0.0027. At P21, p=0.94. **(b)** Oligodendrocytes in the body of the corpus callosum in P45 mice, normalized to callosal volume (*Scn8a*^+/+^ n=8 mice; *Scn8a*^+/mut^ n=6 mice). Two-tailed t-test, p=0.017. Apoptotic OPCs **(c)** and apoptotic mature oligodendrocytes **(d)** in P45 *Scn8a*^+/+^ and *Scn8a*^+/mut^ were labeled with TUNEL staining. **(c)** *Scn8a*^+/+^ n=4 mice, *Scn8a*^+/mut^ n=4 mice, two-tailed t-test: p=0.3471. **(d)** *Scn8a*^+/+^ n=4 mice, *Scn8a*^+/mut^ n=4 mice, two-tailed t-test: p=0.3625. **(e)** Total number of callosal axons (myelinated and unmyelinated) per electron micrograph (field) in *Scn8a*^+/+^ and *Scn8a*^+/mut^ mice. P21: *Scn8a*^+/+^=3 mice; *Scn8a*^+/mut^=3 mice. P45: *Scn8a*^+/+^=4 mice; *Scn8a*^+/mut^=4 mice. One-way ANOVA revealed no significant variance in total axon number among the four groups, F(3, 10)=4.047, p=0.04. Sidak’s post-hoc test, *Scn8*a^+/mut^ vs *Scn8a*^+/+^ at P45, p=0.37. At P21, p=0.13. *Scn8a*^+/+^ mice are indicated by black dots and *Scn8a*^+/mut^ mice are indicated by red dots. Data are shown with group means ± SEM. *p<0.05, **p<0.01, ***p<0.001, n.s.=nonsignificant (p>0.05). Source data

**Extended Data Fig. 7:**
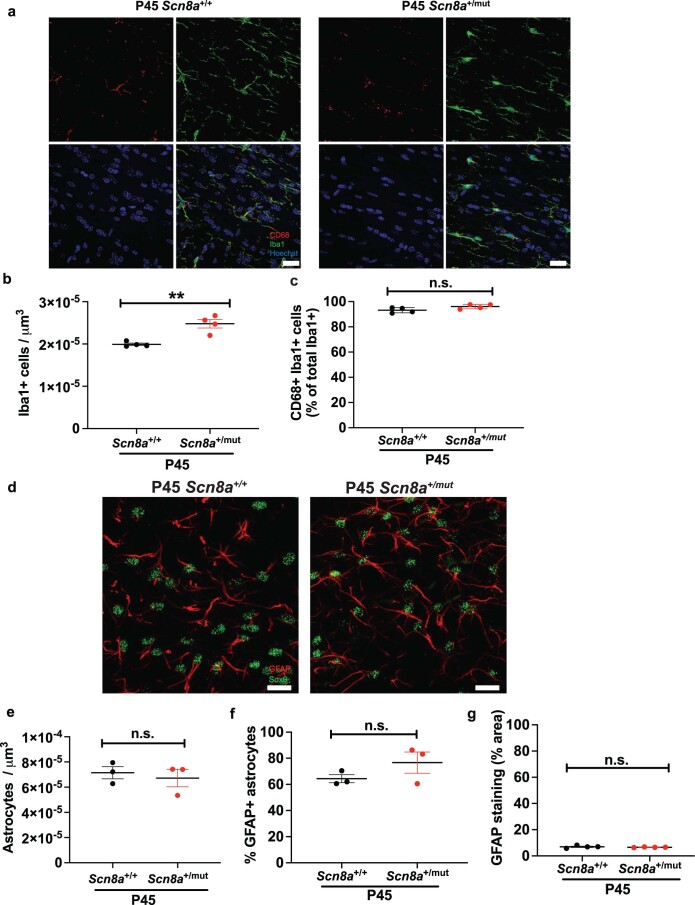
Increased callosal microglial density in *Scn8a*^+/mut^ mice. **(a)** Representative photomicrographs of callosal microglia co-labeled for Iba1 (green), CD68 (red) and nuclear Hoescht staining (blue), from P45 *Scn8a*^+/+^ and *Scn8a*^*+/mut*^ mice. Scale bars=20 μm. **(b)** Density of Iba1-expressing microglia for each mouse. *Scn8a*^+/+^ n=4 mice; *Scn8a*^+/mut^ n=4 mice. Data were analyzed with two-tailed t-test, p=0.0035. **(c)** The percent of Iba1+ microglia co-expressing CD68 appears to be relatively high in C3H/FeJ mice, but was not significantly different in *Scn8a*^+/+^ (n=4 mice) and *Scn8a*^+/mut^ (n=4 mice); two-tailed t-test, p=0.0806. **(d)** Representative photomicrographs of callosal astrocytes co-labeled for Sox9 (green) and GFAP (red), from P45 *Scn8a*^+/+^ and *Scn8a*^*+/mut*^ mice. Scale bars=20 μm. **(e)** Total astrocyte density (GFAP and Sox9- and Sox9-only expressing astrocytes). *Scn8a*^+/+^, n=3 mice and *Scn8a*^+/mut^, n=3 mice; two-tailed t-test, p=0.6390. **(f)** The percentage of GFAP positive astrocytes was equivalent (*Scn8a*^+/+^, n=3 mice and *Scn8a*^+/mut^, n=3 mice; two-tailed t-test, p=0.2286). **(g)** Astrocyte size as assessed by % area occupied by GFAP staining was equivalent between *Scn8a*^+/+^ and *Scn8a*^+/mut^ (*Scn8a*^+/+^, n=4 mice and *Scn8a*^+/mut^, n=4 mice; two-tailed t-test, p=0.4844). Each dot represents the mean for one mouse; group means ± SEM are shown. *Scn8a*^+/+^ mice are shown in black and *Scn8a*^+/mut^ are shown in red. *p<0.05, **p<0.01, ***p<0.001, n.s.=nonsignificant (p>0.05). Source data

Before seizure onset (P21), callosal OPC proliferation and oligodendrocyte number were equivalent in *Scn8a*^+/mut^ mice and littermate wild-type control mice (*Scn8a*^+/+^). In contrast, after seizures are well-established at P45, we found increased proliferating OPCs (*Scn8a*^+/+^: 8,562 ± 592 Ki67^+^ OPCs and *Scn8a*^+/mut^: 11,390 ± 502 Ki67^+^ OPCs; 33% increase) and increased mature oligodendrocytes (*Scn8a*^+/+^: 110,369 ± 20,189 oligodendrocytes and *Scn8a*^+/mut^: 207,716 ± 27,252 oligodendrocytes; 88% increase) in the corpus callosum of *Scn8a*^+/mut^ animals relative to littermate controls (Fig. 3a–d). Noting that corpus callosum volume is increased in *Scn8a*^+/mut^ mice at P45 but not at P21 (Extended Data Fig. 6a), likely due to increased callosal myelination, we normalized oligodendrocyte quantities at P45 to callosal volume to assess cell density. This demonstrated increased mature oligodendrocyte density in the corpus callosum of *Scn8a*^+/mut^ mice compared to *Scn8a*^+/+^ mice (Extended Data Fig. 6b). There was no difference in callosal OPC or oligodendrocyte cell death, assessed with TUNEL staining (Extended Data Fig. 6c,d). Because neuro-inflammation related to microglial and astrocyte reactivity can affect activity-dependent myelination^11,37^, we quantified microglia density and activation state and assessed astrocyte density and hypertrophy. We found a small increase in callosal microglial cell density in P45 *Scn8a*^+/mut^ mice relative to *Scn8a*^+/+^ mice, whereas microglial reactivity (assessed with CD68 immunostaining) was equivalent between P45 *Scn8a*^+/mut^ and *Scn8a*^+/+^ mice (Extended Data Fig. 7a–c). Astrocytes did not exhibit hypertrophy or increased cell density (Extended Data Fig. 7d–g). Taken together, these findings are not suggestive of a pronounced state of microglial or astrocyte reactivity in P45 *Scn8a*^+/mut^ mice.

Transmission electron microscopy revealed that myelin sheath thickness was increased in association with established seizures at P45 in *Scn8a*^+/mut^ mice relative to *Scn8a*^+/+^ littermate controls (*Scn8a*^+/+^ g-ratio: 0.75 ± 0016 and *Scn8a*^+/mut^ g-ratio: 0.71 ± 0.008; Fig. 3e–h). Before seizure onset at P21, g-ratios were equivalent in *Scn8a*^+/mut^ and littermate control mice (Fig. 3f,h). Mean myelinated axon diameter was equivalent at P21 and at P45 in *Scn8a*^+/mut^ mice relative to *Scn8a*^+/+^ littermate controls, indicating that altered axon size does not contribute to g-ratio differences (Extended Data Fig. 2c). We also found an increase in the percent of axons that were myelinated at P45 in *Scn8a*^+/mut^ mice compared to *Scn8a*^+/+^ mice, whereas total axon density was equivalent (Fig. 3i and Extended Data Fig. 6e). Similar to Wag/Rij rats, we did not observe that increased myelin sheath thickness in *Scn8a*^+/mut^ mice was restricted to any particular axon size (Extended Data Fig. 4c,d).

Taken together, findings in Wag/Rij rat and *Scn8a*^+/mut^ mouse models demonstrate that absence seizures induce increased OPC proliferation and increased oligodendrocyte generation together with abnormally increased myelination within the affected thalamocortical seizure network.

We next sought to determine the functional effect of seizure-associated myelination and tested the hypothesis that aberrantly increased myelination contributes to disease pathogenesis.

### Myelin plasticity promotes generalized epilepsy progression

In the healthy brain, activity-dependent myelination is thought to promote coordination between regions within distributed neuronal networks, a process that supports multiple forms of learning^7,11–17^. We hypothesized that absence seizure-associated, abnormally increased myelination might contribute to thalamocortical network hypersynchrony^30,32^, increasing disease severity. To assess the functional effect of myelin plasticity in absence seizure progression, we sought to block activity-dependent myelination. Activity-dependent secretion of brain-derived neurotrophic factor (BDNF), and its subsequent signaling through the TrkB receptor on OPCs, is required for activity-dependent myelination of corticocallosal projection neurons^11^. Conditional deletion of TrkB from OPCs prevents activity-dependent oligodendrogenesis and myelination in the corpus callosum but does not alter homeostatic oligodendrogenesis nor lead to myelin loss^11^.

To enable blockade of activity-dependent myelination in *Scn8a*^+/mut^ mice, we bred *Scn8a*^+/mut^ and *Scn8a*^+/+^ littermates with floxed *TrkB* in the presence or absence of Cre, inducibly expressed under the *PDGFRα* promoter (*Scn8a*^+/mut^;*TrkB*^fl/fl^; *PDGFRα::Cre-ER*). Induction of Cre in this model with tamoxifen leads to TrkB deletion in about 80% of OPCs^11^; leak of Cre expression is not found in neurons^38^. Our cross yielded four littermate groups of mice: (1) *Scn8a*^+/+^;*TrkB*^fl/fl^ (referred to as *Scn8a*^+/+^, wild-type mice with intact activity-dependent myelination); (2) *Scn8a*^+/+^;*TrkB*^fl/fl^;*PDGFRα*::*Cre-ER* (referred to as *Scn8a*^+/+^ OPC conditional knockout (cKO), wild-type mice with impaired activity-dependent myelination); (3) *Scn8a*^+/mut^;*TrkB*^fl/fl^ (referred to as *Scn8a*^+/mut^, mice with absence seizures and intact activity-dependent myelination); and (4) *Scn8a*^+/mut^;*TrkB*^fl/fl^;*PDGFRα*::*Cre-ER* (*Scn8a*^+/mut^ OPC cKO, mice with absence seizures and impaired activity-dependent myelination). All mice were treated with tamoxifen to ensure that any differences between genotype groups do not reflect differences in tamoxifen treatment. After tamoxifen treatment, mice were implanted for EEG to monitor seizures.

**Extended Data Fig. 8:**
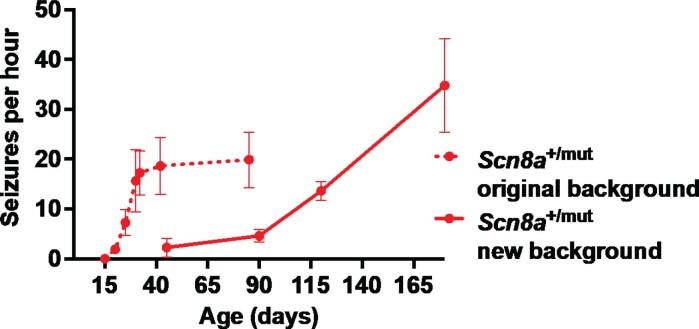
Timing and kinetics of seizure onset and epilepsy progression in *Scn8a*^+/mut^ mice on original (C3HeB/FeJ) and new (C3HeB/FeJ/C57/BL6) genetic backgrounds. To genetically block activity-dependent myelination in *Scn8a*^+/mut^ mice (originally on a C3HeB/FeJ background), we generated *Scn8a*^+/mut^;*TrkB*^fl/fl^;*PDGFRα::Cre-ER* mice (on a C3HeB/FeJ and C57/BL6 mixed genetic background). In *Scn8a*^+/mut^ mice with the new mixed C3HeB/FeJ and C57/BL6 genetic background and intact activity-dependent myelination (*Scn8a*^+/mut^, new background), seizure onset and progression occurred at later timepoints (solid red line, P45-P180, 1.5- to 6-months, P45, n=4 mice, P90, n=6 mice, P120, n=6 mice, P180, n=5 mice). In the original line (*Scn8a*^+/mut^, original background), seizures begin at ~P21 and increase until P35-P45 (dashed red line, n=4 mice per timepoint, data from Makinson et al, *Neuron* 2017^37^). Graph displays group means with SEM. Source data

The original *Scn8a*^*+/mut*^ mouse line is on a C3HeB/FeJ background, whereas *Scn8a*^*+/mut*^;*TrkB*^*fl/fl*^ mice have a mixed C3HeB/FeJ and C57/BL6 background. Background strain can influence the age of seizure onset and progression^39^. Therefore, we determined the timeline of epileptogenesis in *Scn8a*^*+/mut*^ mice with this mixed background. In *Scn8a*^*+/mut*^ mice (mixed background) with intact activity-dependent myelination, 4–8-Hz absence seizures begin around P90. Seizures then increase steadily and occur 20–30 times per hour, on average, by 6 months of age (Extended Data Fig. 8).

**Fig. 4:**
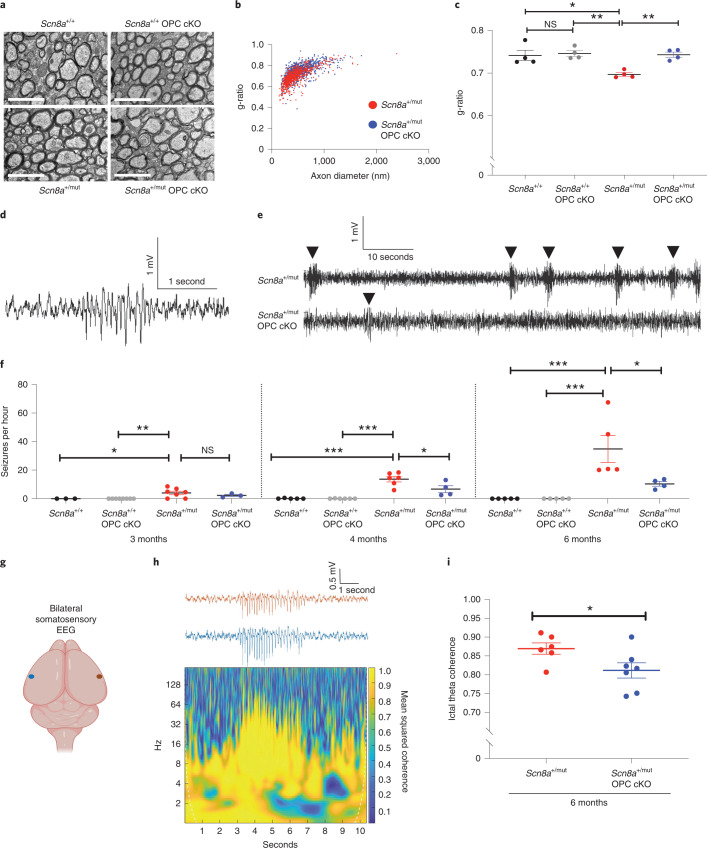
Activity-dependent myelination contributes to generalized epilepsy progression. **a**, Representative transmission electron micrographs from corpus callosum body of 6-month-old mice. Scale bar, 2 μm. **b**, g*-*ratios from 6-month-old *Scn8a*^+/mut^ and *Scn8a*^+/mut^ OPC cKO mice. Each dot represents the g-ratio for one axon. **c**, Mean g-ratios for each mouse. *Scn8a*^+/+^, *n* = 4 mice; *Scn8a*^+/+^ OPC cKO, *n* = 4 mice; *Scn8a*^+/mut^, *n* = 4 mice; *Scn8a*^+/mut^ OPC cKO, *n* = 4 mice. One-way ANOVA, *F*_3,12_ = 8.753, *P* = 0.0024. Post hoc Tukey’s test: *Scn8a*^+/mut^ versus *Scn8a*^+/+^, *P* = 0.0085, *Scn8a*^+/mut^ versus *Scn8a*^+/+^ OPC cKO, *P* = 0.0042. *Scn8a*^+/mut^ OPC cKO mice versus *Scn8a*^+/+^, *P* > 0.99. *Scn8a*^+/mut^ OPC cKO mice versus *Scn8a*^+/+^ OPC cKO, *P* > 0.99. *Scn8a*^+/mut^ OPC cKO versus *Scn8a*^+/mut^ mice, *P* = 0.0066. *Scn8a*^+/+^ versus *Scn8a*^+/+^ OPC cKO, *P* = 0.9756. **d**, Representative seizure in a *Scn8a*^+/mut^ mouse. **e**, Continuous EEG recordings showing decreased incidence of seizures (arrowheads) in *Scn8a*^+/mut^ OPC cKO mice at 6 months. **f**, Mean seizures per hour for each mouse. 3 months: *Scn8a*^+/+^, *n* = 3 mice; *Scn8a*^+/+^ OPC cKO, *n* = 8 mice; *Scn8a*^+/mut^, *n* = 7, *Scn8a*^+/mut^ OPC cKO, *n* = 3 mice. One-way ANOVA: *F*_3,17_ = 5.814, *P* = 0.0063. Post hoc Tukey’s test: *Scn8a*^+/+^ versus *Scn8a*^+/mut^, *P* = 0.045. *Scn8a*^+/mut^ versus *Scn8a*^+/mut^ OPC cKO, *P* = 0.6. 4 months: *Scn8a*^+/+^, *n* = 5 mice; *Scn8a*^+/+^ OPC cKO, *n* = 6 mice; *Scn8a*^+/mut^, *n* = 6 mice, *Scn8a*^+/mut^ OPC cKO, *n* = 4 mice. One-way ANOVA: *F*_3,17_ = 23.05, *P* < 0.0001. Tukey’s test: *Scn8a*^+/+^ versus *Scn8a*^+/mut^, *P* < 0.0001. *Scn8a*^+/mut^ versus *Scn8a*^+/mut^ OPC cKO, *P* = 0.0193. 6 months: *Scn8a*^+/+^, *n* = 5 mice; *Scn8a*^+/+^ OPC cKO, *n* = 5 mice; *Scn8a*^+/mut^, *n* = 5 mice; *Scn8a*^+/mut^ OPC cKO, *n* = 4 mice. One-way ANOVA: *F*_3,15_ = 11.13, *P* = 0.0004. Tukey’s test: *Scn8a*^+/+^ versus *Scn8a*^+/mut^, *P* = 0.0008. *Scn8a*^+/mut^ versus *Scn8a*^+/mut^ OPC cKO, *P* = 0.0218. **g**, Schematic of recording electrodes over somatosensory cortices, created with BioRender. **h**, Representative seizure from a 6-month-old *Scn8a*^+/mut^ mouse with coherence plot. **i**, Ictal theta band coherence. *Scn8a*^+/mut^, *n* = 6 mice, *Scn8a*^+/mut^ OPC cKO, *n* = 7 mice. Two-tailed *t*-test: *P* = 0.047. Each dot represents the mean for one animal (**c**, **f**, **i**), with group means ± s.e.m. *Scn8a*^+/+^, black dots; *Scn8a*^+/+^ OPC cKO, gray dots; *Scn8a*^+/mut^, red dots; *Scn8a*^+/mut^ OPC cKO, blue dots. *P < 0.05, **P < 0.01, ***P < 0.001, NS, non-significant (*P* > 0.05). Source data

We next confirmed that deletion of the TrkB receptor from OPCs prevents the myelination response to seizures. As expected, deletion of the TrkB receptor from OPCs in *Scn8a*^*+/mut*^;*TrkB*^*fl/fl*^;*PDGFRα::Cre* mice (*Scn8a*^*+/mut*^ OPC cKO) prevented the aberrantly increased myelination (decreased g-ratio) observed in *Scn8a*^+/mut^ mice (Fig. 4a–c). Differences in g-ratios were not related to changes in axonal diameter (Extended Data Fig. 2d).

**Extended Data Fig. 9:**
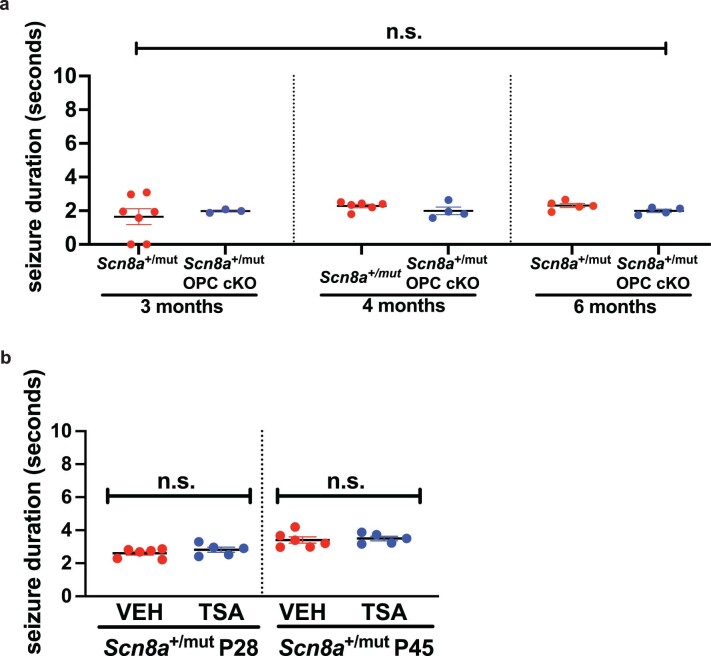
Activity-dependent myelination does not impact seizure duration. **(a)** Mean seizure duration in *Scn8a*^+/mut^ (red dots) and *Scn8a*^+/mut^ OPC cKO mice (blue dots). 3-month-old timepoint: *Scn8a*^+/mut^ n=7 mice, *Scn8a*^+/mut^ OPC cKO, n=3 mice. 4-month-old timepoint: *Scn8a*^+/mut^, n=6 mice, *Scn8a*^+/mut^ OPC cKO, n=4 mice. 6-month timepoint: *Scn8a*^+/mut^, n=5 mice, *Scn8a*^+/mut^ OPC cKO, n=4 mice. One-way ANOVA, F(5, 23)=0.7687, p=0.58. **(b)** Mean seizure duration in *Scn8a*^+/mut^ treated with vehicle (VEH, red dots) or trichostatin A (TSA, blue dots). P28 timepoint: *Scn8a*^+/mut^-VEH n=6 mice, *Scn8a*^+/mut^-TSA n=5 mice. P45 timepoint: *Scn8a*^+/mut^-VEH n=6 mice, *Scn8a*^+/mut^-TSA n=5 mice. Data were analyzed with one-way ANOVA revealing significant variation in seizure duration across groups, F(3,18)=8.062, p=0.0013. However, Sidak’s test comparing seizure duration between groups within each timepoint revealed no significant differences (P28, p=0.57; P45, p=0.90). Each data point represents mean seizure duration for one mouse; data are shown with group means ± SEM. n.s., nonsignificant (p>0.05). Source data

Having elucidated the timeline of seizure progression and confirmed that TrkB deletion from OPCs prevents aberrant myelination in association with seizures, we next examined the number of seizures per hour in *Scn8a*^*+/mut*^ mice lacking TrkB expression in OPCs (*Scn8a*^*+/mut*^ OPC cKO). We found that seizure burden was strikingly reduced in *Scn8a*^*+/mut*^ OPC cKO mice with impaired activity-dependent myelination. *Scn8a*^*+/mut*^ mice with intact activity-regulated myelination exhibit a marked increase in the number of seizures per hour over time (Fig. 4d–f). In contrast, *Scn8a*^*+/mut*^ mice with OPC-specific loss of TrkB expression that lack activity-regulated myelination exhibit substantially fewer seizures per hour, an effect that was sustained at least until 6 months of age (4-month time point: *Scn8a*^*+/mut*^ seizures per hour, 13.64 ±1.88, and *Scn8a*^*+/mut*^ OPC cKO seizures per hour, 6.7 ± 2.45, a 51% decrease; 6-month time point: *Scn8a*^*+/mut*^ seizures per hour, 34.83 ± 9.45, and *Scn8a*^*+/mut*^ OPC cKO seizures per hour, 10.34 ± 1.67, a 70% decrease. Data are shown in Fig. 4f). The mean duration of individual seizures in *Scn8a*^+/mut^ mice was 2.3 ± 0.2 seconds, consistent with previously published findings in *Scn8a*^+/mut^ mice^30^; the mean duration of individual seizures was not significantly different in *Scn8a*^+/mut^ cKO mice (Extended Data Fig. 9a). Taken together, these findings indicate that activity-dependent myelination contributes to increasing absence seizure burden over time.

### Myelin plasticity promotes ictal synchrony

**Extended Data Fig. 10:**
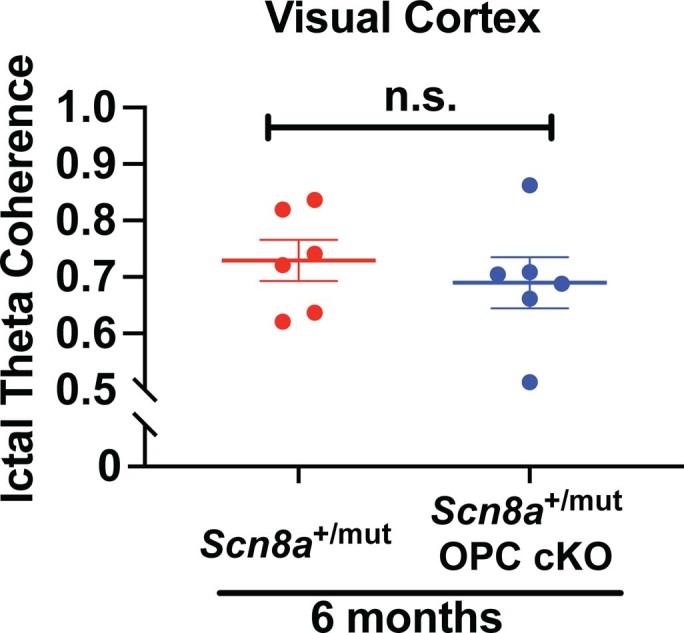
Blockade of activity-dependent myelination does not alter ictal coherence in visual cortices. In contrast to ictal coherence in the somatosensory cortices (Fig. 4i), ictal theta band coherence when recorded from visual cortices is similar in 6-month-old *Scn8a*^+/mut^ OPC cKO mice compared to *Scn8a*^+/mut^ mice. *Scn8a*^+/mut^ (red dots), n=6 mice, *Scn8a*^+/mut^ OPC cKO (blue dots), n=6 mice. Each dot represents the mean ictal theta coherence from visual cortices for one mouse, shown with group means ± SEM. Two-tailed t-test, p=0.5142. n.s., nonsignificant (p>0.05). Source data

Mechanisms of seizure genesis are thought to originate in intrinsic neuronal properties, independent from myelination^30,32^, raising the question of how changes in myelination might contribute to seizure burden. We hypothesized that aberrant myelination might promote seizure progression by further facilitating ictal synchrony once the thalamocortical network has already become prone to seizures. To test this idea, we assessed inter-hemispheric somatosensory cortical ictal coherence with EEG in 6-month-old *Scn8a*^+/mut^ and *Scn8a*^+/mut^ OPC cKO mice, focusing on theta frequency activity (4–8 Hz). We found that ictal EEG coherence between the somatosensory cortices was substantially diminished in *Scn8a*^+/mut^ OPC cKO mice that lack activity-dependent myelination, compared to *Scn8a*^+/mut^ mice (Fig. 4g–i). By contrast, there was no difference in ictal coherence in the bilateral visual cortices (Extended Data Fig. 10), where seizure activity is minimal and where myelin changes were not observed in Wag/Rij rats (Extended Data Fig. 5).

### Blockade of myelin plasticity abrogates epilepsy progression

**Fig. 5:**
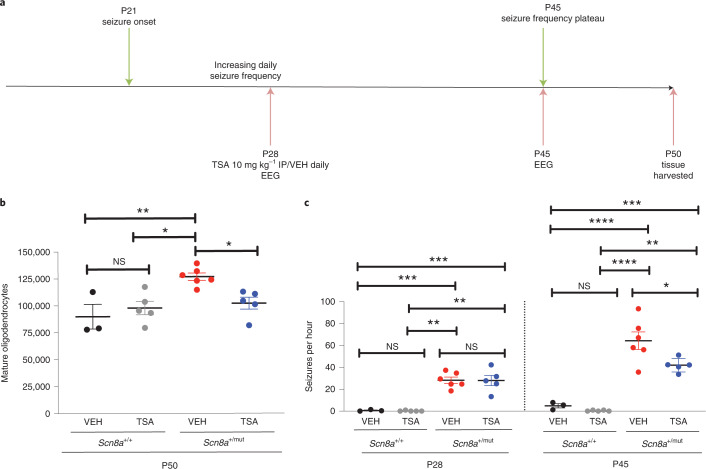
Pharmacological blockade of oligodendrogenesis decreases generalized epilepsy progression. **a**, Vehicle (VEH) or TSA HDAC inhibitor treatments were initiated at P28 (~1 week after seizure onset); EEG was recorded at P28 and P45. **b**, Total callosal oligodendrocytes for each mouse. *Scn8a*^+/+^-VEH, *n* = 3 mice; *Scn8a*^+/+^-TSA, *n* = 5 mice; *Scn8a*^+/mut^-VEH, *n* = 6 mice, *Scn8a*^+/mut^-TSA, *n* = 5 mice. One-way ANOVA: *F*_3,15_ = 7.433, *P* = 0.0028. Post hoc Tukey testing: VEH-treated *Scn8a*^+/mut^ mice oligodendrocyte number versus *Scn8a*^+/+^-VEH (*P* = 0.0055) and *Scn8a*^+/+^-TSA (*P* = 0.011); TSA-treated *Scn8a*^+/mut^ mice had fewer mature oligodendrocytes than *Scn8a*^+/mut^ VEH-treated mice (*P* = 0.031), equivalent to *Scn8a*^+/+^-VEH (*P* = 0.59). **c**, Mean seizures per hour for each mouse. P28 time point: *Scn8a*^+/+^-VEH, *n* = 3 mice; *Scn8a*^+/+^-TSA *n* = 5 mice; *Scn8a*^+/mut^-VEH *n* = 6 mice; *Scn8a*^+/mut^-TSA *n* = 5 mice. One-way ANOVA: *F*_3,15_ = 25.95, *P* < 0.0001. Tukey testing at P28 showed no pre-treatment difference in seizure burden (seizures per hour) between *Scn8a*^+/mut^-VEH and *Scn8a*^+/mut^-TSA groups (*P* > 0.99). *Scn8a*^+/mut^-VEH versus *Scn8a*^+/+^-VEH, *P* = 0.0002; *Scn8a*^+/mut^-VEH versus *Scn8a*^+/+^-TSA, *P* < 0.0001; *Scn8a*^+/mut^-TSA versus *Scn8a*^+/+^-VEH, *P* = 0.0003; *Scn8a*^+/mut^-TSA versus *Scn8a*^+/+^-TSA, *P* < 0.0001. P45 time point: *Scn8a*^+/+^-VEH *n* = 3 mice; *Scn8a*^+/+^-TSA *n* = 5 mice; *Scn8a*^+/mut^-VEH *n* = 6 mice; *Scn8a*^+/mut^-TSA *n* = 5 mice. One-way ANOVA: *F*_3,15_ = 33.25, *P* < 0.0001. Tukey testing revealed increased seizures in *Scn8a*^+/mut^ groups (*Scn8a*^+/mut^-VEH versus *Scn8a*^+/+^-VEH, *P* < 0.0001; *Scn8a*^+/mut^-VEH versus *Scn8a*^+/+^-TSA, *P* < 0.0001; *Scn8a*^+/mut^-TSA versus *Scn8a*^+/+^-VEH, *P* = 0.0032; *Scn8a*^+/mut^-TSA versus *Scn8a*^+^^/+^-TSA, *P* = 0.0003); however, *Scn8a*^+/mut^-TSA treated mice had fewer seizures than *Scn8a*^+/mut^-VEH (*P* = 0.032). *Scn8a*^+/+^-VEH versus *Scn8a*^+/+^-TSA: P28, *P* > 0.99; P45, *P* = 0.95. Each dot represents the mean for one mouse; group means ± s.e.m. are shown. *Scn8a*^+/+^-VEH, black dots; *Scn8a*^+/+^-TSA, gray dots; *Scn8a*^+/mut^-VEH, red dots; *Scn8a*^+/mut^-TSA, blue dots. *P < 0.05, **P < 0.01, ***P < 0.001, NS, non-significant (*P* > 0.05). Source data

To further assess the role of activity-dependent myelination in seizure progression, and to assess the potential for targeting maladaptive myelination in the treatment of absence seizures, we next pharmacologically blocked activity-dependent myelination in *Scn8a*^+/mut^ mice on the C3HeB/FeJ background (with seizure onset at P21), to assess the effect on seizure burden. Trichostatin A (TSA) is a histone deacetylase (HDAC) inhibitor that is known to prevent activity-dependent oligodendrogenesis and myelination by its interference in epigenetic changes that are required for the maturation of OPCs to myelinating oligodendrocytes^4^. To simulate a clinically relevant scenario in which therapeutics are administered after seizure onset in humans, we administered TSA daily beginning ~7 days after seizure onset, at P28 and continuing throughout the remaining period of seizure progression (Fig. 5a). We confirmed that TSA administration prevented abnormally increased callosal oligodendrogenesis in *Scn8a*^+/mut^ mice (Fig. 5b). We next assessed the effect of pharmacological inhibition of activity-dependent myelination with TSA on seizure progression. Before treatment initiation on P28, seizure burden in vehicle-treated and TSA-treated *Scn8a*^+/mut^ mice was equivalent (Fig. 5c). However, TSA treatment between P28 and P45 led to decreased seizure burden at P45 (vehicle-treated *Scn8a*^+/mut^ mice: 64.33 ± 8.00; TSA-treated *Scn8a*^+/mut^ mice, 42 ± 2.79, a ~35% decrease in the number of seizures, shown in Fig. 5c) without affecting seizure duration (Extended Data Fig. 9b), similar to genetic blockade of activity-dependent myelination described above.

## Discussion

Given that repetitive patterns of neuronal activity can regulate myelination, and that activity-dependent myelination can influence neural network function^4–9,12–15,17^, we reasoned that aberrant patterns of neuronal activity might induce oligodendrogenesis and myelination within seizure networks. Aberrant myelination could then reciprocally contribute to subsequent maladaptive network plasticity and epilepsy progression. Here, we found network-specific increased oligodendrogenesis and myelination only after the onset of absence seizures in two distinct rodent models (Wag/Rij rats and *Scn8a*^+/mut^ mice) of generalized epilepsy. Increased myelination did not occur when seizures were pharmacologically prevented. Blocking activity-regulated myelination genetically or pharmacologically reduced ictal coherence within the seizure network and abrogated epilepsy progression, preventing the increase in seizure quantity that typically occurs over time in mice with intact myelin plasticity. Taken together, these findings illustrate maladaptive myelination that contributes to disease severity in models of generalized epilepsy.

Absence seizures result from abnormal, hypersynchronous oscillations in thalamocortical connections and generalize to involve both hemispheres of the brain by propagating across the corpus callosum^28^. Multiple epilepsy syndromes, including some that are lifelong and/or medically intractable, involve typical or atypical absence seizures. This includes the generalized genetic epilepsies and the often devastating developmental and epileptic encephalopathy Lennox–Gastaut syndrome^25^. Progressive forms of generalized epilepsy, and historical accounts of the natural history of untreated generalized epilepsy, involve increases in the number and severity of seizures over time, similar to what is seen in animal models^25,31^. Recent evidence indicates that this period of seizure progression is a key determinant of disease severity in humans as well as rodent models, such that early blockade of absence seizures and/or their downstream effects mitigates morbidity^29,40^. Thus, improved understanding of mechanisms underlying seizure progression could enable discovery of disease-modifying and/or curative treatments for generalized and other forms of epilepsy.

The findings presented here indicate that maladaptive myelination is a consequence of seizures and/or related changes in neural activity and does not precede the onset of recurrent seizures in the models that we studied, underscoring the primary role of neurons in epilepsy onset^30,32^. Our findings contrast with, but do not exclude, the possibility that, in some cases, developmental myelin differences preceding seizures, including within the corpus callosum, could also influence seizure severity^41–43^.

Both Wag/Rij rats and *Scn8a*^+/mut^ mice exhibit increased myelin sheath thickness (decreased g-ratio), whereas *Scn8a*^+/mut^ mice also exhibit an increase in myelinated axons, indicative of de novo myelination. These differences may be related to species and/or age of onset of seizures, which is ~2 months in Wag/Rij rats and ~P21 in *Scn8a*^+/mut^ mice. The observed increases in callosal myelin sheath thickness were subtle, but this magnitude of change could substantially modulate network function given the dynamic range of g-ratios^4,6,11,35^. Small changes within this range can meaningfully modulate neuronal network function and behavior^4,11^. Whether the change in myelinated axon number reflects de novo myelination of previously unmyelinated axons or discontinuously myelinated axon segments^44^, and whether thicker myelin sheaths reflect newly generated internodes or activity-regulated remodeling by existing oligodendrocytes^9^, remain to be determined. Furthermore, it will be important to determine whether myelin internode length changes in association with seizures using models amenable to such measurements^10^. The effects of seizure-associated myelination on inhibitory interneurons and other neuronal subtypes remain to be explored^10^.

The observed myelination-dependent increase in ictal somatosensory EEG coherence suggests that aberrantly increased myelination within the thalamocortical network may increase the potential for highly synchronous activity that underlies absence seizures, thus enabling more frequent transition of the network to the seizure state. Activity-regulated myelination may also contribute to epilepsy by influencing spike timing-dependent synaptic plasticity, temporal dynamics involving interneuron function and neuronal excitability^41,42,45,46^ and/or by serving as a compensatory mechanism that provides metabolic support to enable rapid firing during seizures^47^.

Notably, HDAC inhibition has been shown to promote synaptic plasticity^48^; therefore, the observed effects of TSA treatment are unlikely to be explained by impaired synaptic plasticity. HDAC inhibition has also been shown to improve the course of absence epilepsy when initiated before seizure onset in Wag/Rij rats^49^, although the link to oligodendrogenesis has not been previously appreciated.

Our studies also suggest a link between BDNF signaling in OPCs and aberrant myelination that promotes seizures. The role of BDNF signaling in epilepsy is complex (Supplementary Note 1). An important consideration for our study is that BDNF to TrkB signaling was prevented specifically in OPCs (in *Scn8a*^+/mut^ OPC cKO mice). Thus, we did not determine the effect of BDNF signaling blockade across all cell types (such as neurons) on seizures, nor do we conclude that BDNF antagonism would be a useful therapeutic for the treatment of absence seizures. Future work should explore whether additional molecular pathways that link neurons, oligodendrogenesis and myelination are involved in epilepsy (Supplementary Note 1).

The broader implications of these findings in rodent models to generalized epilepsy in humans remain to be fully elucidated, and several open questions remain for future study (Supplementary Note 2). Given the many mechanisms occurring in different human forms of epilepsy (including differences in age at onset, seizure location, seizure severity, etiology and associated neuro-inflammation), it is likely that the extent and role of myelin plasticity also varies between different types of epilepsy.

Mounting evidence suggests that a range of aberrant patterns of myelination may predispose brain networks toward seizures, through multiple mechanisms. Our findings in models of generalized epilepsy indicate that activity-regulated myelination, previously described in the setting of neural network adaptation related to learning, can also reinforce deleterious patterns of neural activity. Therefore, myelin plasticity may become maladaptive in some contexts. More myelin is not necessarily better, and seizure-related plasticity that increases myelination of axons beyond a normal optimum could disrupt the normal function, for example by interfering with precise oscillatory synchrony between brain regions that supports cognition^7,12–14^. This raises a key question about how oligodendrocyte lineage cells may sense and integrate circuit-level information to fine-tune circuit dynamics in an optimal manner in health. Further study of maladaptive myelination in disease contexts may elucidate novel strategies to treat neurological diseases, such as epilepsy, while also providing greater insight into mechanisms of myelin plasticity that promote function in the healthy brain.

## Methods

### Rodent colony maintenance

All experiments were conducted in accordance with protocols approved by the Stanford University Institutional Animal Care and Use Committee (protocols 27215, 12363 and 33969). Mice or rats were group or single housed (up to five mice or two rats per cage) according to standard guidelines, with ad libitum access to food and water on a 12-hour light/dark cycle. In rooms where mice and rats were housed, the ambient temperature was 70 ± 2° F, and the relative humidity was 30–70%. No animals were manipulated other than as reported for that experimental group—that is, there was no history of drug exposures, surgeries or behavioral testing for the animals used other than that reported for the given experimental group. Mice and rats were healthy and tolerated all experimental manipulations well.

The ages of all mice and rats used in specific studies are indicated in the figures and throughout the text. In brief, in studies using Wag/Rij and control rats, 1.5-month-old animals were used to assess endpoints before seizure onset, and 6–7-month-old animals were used to assess endpoints after seizures are well-established. In studies using *Scn8a*^+/mut^ mice and wild-type littermates, P21 mice were used to assess endpoints before seizure onset, whereas P45 mice were used to assess endpoints after seizures are well-established. In studies in which *Scn8a*^+/mut^ and other mouse lines were bred (Fig. 4), because seizure onset is delayed, later time points (3–6 months) were used to study seizure progression, as described in detail in the text and methods below.

Both males and females (rats and mice) were used in equal numbers whenever possible. The exact numbers of male and female rats and mice are listed in the source files. There was no discernable effect of male or female sex upon any of the endpoints, aside from body weight. For most experiments, individual animals used came from at least 2–3 distinct litters.

### Wag/Rij rats

Wistar (control) and Wag/Rij rats were purchased from Charles Rivers Laboratories (Wistar: cat. no. 003; Wag/Rij, Charles Rivers Italy: strain code 638). A colony of Wag/Rij rats has subsequently been maintained in the laboratory of J.R.H. at Stanford^50^.

### *Scn8a*^+/mut^ mice and *Scn8a*^+/mut^ OPC cKO mice

*Scn8a*^+/mut^ mice with the *med* loss-of-function mutation in *Scn8a* (C3Fe.Cg-Scn8amed/J, jax.org/strain/003798)^30,36^ were bred to wild-type mice (*Scn8a*^+/+^) on a congenic background strain (C3HeB/FeJ, jax.org/strain/00658). In separate studies, successive breeding was performed to obtain mice with absence seizures (*Scn8a*^+/mut^) and inducible deletion of the TrkB receptor from OPCs, enabling blockade of activity-dependent myelination (*Scn8a*^+/mut^;*TrkB*^fl/fl^;*PDGFRα*::*Cre*-ER)^11^. Specifically, *Scn8a*^+/mut^ mice on the C3HeB/FeJ background were crossed with mice expressing a ‘floxed’ *TrkB* gene (*TrkB*^fl/fl^) on a C57/BL6 background to obtain *Scn8a*^+/mut^ and *Scn8a*^+/+^ mice with floxed *TrkB* (*Scn8a*^+/mut^;*TrkB*^fl/fl^ or *Scn8a*^+/+^;*TrkB*^fl/fl^). These mice were crossed with mice with floxed *TrkB* and tamoxifen-inducible *Cre* under the *PDGFRα* promotor (*TrkB*^fl/fl^;*PDGFRα*::*Cre*-ER), also on a C57/BL6 background. Both the *PDGFRα*::*Cre*-ER (originally acquired from Jackson Laboratory, jax.org/strain/018280) and *TrkB*^fl/fl^ (originally acquired from the Mutant Mouse Regional Resource Centers at the University of California, Davis, strain 033048-UCD) mouse lines are maintained in our laboratory and are described in previously published work^11^. Mice were treated with tamoxifen (Sigma-Aldrich, T5648), 100 mg kg^−1^ intraperitoneally for 3 days (P21–P23), which induces robust *Cre* expression and deletion of the TrkB receptors from OPCs in *TrkB*^fl/fl^;*PDGFRα*::*Cre*-ER mice but not mice lacking *Cre* (*TrkB*^fl/fl^ only)^11^. In later experiments in which EEG coherence was assessed, tamoxifen was administered closer to the time of seizure onset, between P75 and P77.

### Genotyping

At P10, mice were ear and/or tail clipped, and DNA was extracted using the REDExtract-N-Amp Tissue PCR Kit (Sigma-Aldrich, XNAT-100RXN). A master mix of REDEX reagent, primers as indicated below, water and extracted DNA from each animal were created for a total volume of 20 µl for each animal. For detection of the floxed *TrkB* allele, the following primers (acquired from IDT) were used: *TrkB fl/fl* F: ATGTCGCCCTGGCTGAAGTG; *TrkB fl/fl* R: ACTGACATCCGTAAGCCAGT. The polymerase chain reaction (PCR) protocol was 94 °C for 3 minutes × 1; then 94 °C for 1 minute, 65 °C for 1.5 minutes and 72 °C for 1.5 minutes, all × 40 cycles; then 72 °C for 7 minutes × 1; then held at 12 °C until used in gel electrophoresis. The PCR products were run on a 1.8% agarose gel, and a band size of 450 base pairs (bp) indicated the presence of the floxed allele. For detection of *Cre*, the following primers (IDT) were used: *Cre* Up: GAT CTC CGG TAT TGA AAC TCC AGC; *Cre* Down: GCT AAA CAT GCT TCA TCG TCG G. PCR protocol was 94 °C for 5 minutes × 1; 94 °C × 15 seconds, 65 °C × 30 seconds, dropping 1 °C after each cycle, 72 °C for 40 seconds, all × ten cycles; then 94 °C for 15 seconds, 55 °C for 30 seconds, 72 °C for 40 seconds, all × 29 cycles; then 72 °C for 2 minutes × 1; then held at 4 °C until used in gel electrophoresis. The presence of a 650-bp PCR product on a 1.8% agarose gel indicated an animal positive for the *Cre* gene. Automated genotyping for the *Scn8a* ‘med’ allele was done using tail samples via Transnetyx (www.transnetyx.com).

### ETX treatment

Wistar (control) and Wag/Rij rats were treated with ETX or vehicle on a previously published dosing schedule^29^ shown to prevent and/or substantially reduce seizures. Male and female littermates were randomly assigned to either vehicle or ETX treatment. Pharmaceutical-grade ETX solution (Akorn Pharmaceuticals, 250 mg ml^−1^, NDC: 61748-024-16) was added to drinking water in bottles that were shielded from light at a concentration of 2.5 mg ml^−1^, leading to approximate dosing of 300 mg/kg/day^29^. The solution was changed at least every 7 days, and the health, weight and drinking of animals were monitored regularly throughout the study. Before perfusion, 1–2 ml of blood was taken. The samples were centrifuged at 2,000*g*, 4 °C, for 5 minutes, and plasma was collected for measurement of ETX concentration using liquid chromatography–mass spectroscopy, performed at the Stanford BioADD center (http://med.stanford.edu/bioadd.html).

### TSA treatment

*Scn8a*^*+/+*^ and *Scn8a*^*+/mut*^ mice were treated with the HDAC inhibitor TSA or vehicle on a previously published dosing schedule shown to prevent activity-dependent myelination in mice^11^. Male and female littermates were randomly assigned to either vehicle or TSA treatment. TSA (Selleck Chemicals, S1045) stock solution (50 mg ml^−1^ in sterile-filtered DMSO) was kept at −20 °C until the day of dosing, when it was diluted to a concentration of 10 mg ml^−1^ in 30% PEG300 (Selleck Chemicals, S6704) and 2% Tween 80 (Sigma-Aldrich, P1754) in sterile-filtered DMSO (Tocris, 3176) just before each administration. Then, 10 mg kg^−1^ of TSA or vehicle was administered each day by intraperitoneal injection^4^ between P28 (starting after the first EEG recording on the same date) and P45. Mice were weighed every 1–2 days, and their health was monitored throughout the study. We did not observe any deleterious effects of TSA treatment.

### EEG

Mice and rats were stereotactically implanted with wires (A-M Systems, 791400) soldered to screws (J.I. Morris, FF00CE125) over the bilateral somatosensory cortices as well as a reference wire over cerebellum, and implants were secured with dental cement (Metabond, S399, S371 and S398; also Jet Set4 Liquid, Lang Dental, 3802X6). The following stereotactic coordinates were used, relative to bregma: primary somatosensory cortex (S1BF), AP −1.3 mm and lateral 3.3 mm; primary visual cortex (V1), AP −3.3 mm, lateral 2.5 mm. Implanted wires were integrated into custom-made Mill-Max headpieces (Digi-Key Electronics, ED90267-ND) that could be connected to a head stage, consisting of a digitizer and amplifier board (Intan Technologies, C3334). Awake and freely behaving animals were tethered to an acquisition board (Open Ephys) with lightweight SPI interface cables (Intan Technologies, C3206). Continuous real-time EEG was recorded with Open Ephys software (https://open-ephys.org, version 0.4.4.1). Data were sampled at 2 kHz and bandpass filtered between 1 Hz and 300 Hz. All animals underwent 3–4 hours of continuous EEG recording between the hours of 10:00 and 18:00. Longitudinal data were collected whenever possible (for example, when not precluded by EEG implant loss or failure).

### Immunohistochemistry

Rats were given a lethal dose of Fatal Plus (sodium pentobarbital, Vortech Pharmaceuticals, NDC: 0298-9373-68) and transcardially perfused with 40–80 ml of ice-cold 0.1 M PBS, followed by 40–80 ml of 4% paraformaldehyde (PFA) in PBS before brain extraction and tissue processing. Mice were given a lethal dose of Fatal Plus or 2.5% Avertin (2,2,2-tribromoethanol, Sigma-Aldrich, T48402) and transcardially perfused with 10–20 ml of ice-cold PBS, followed by 10–20 ml of 4% PFA in PBS. In TSA studies, brains were bisected in the sagittal plane at the inter-hemispheric fissure. Brains or hemi-brains were post-fixed in 4% PFA overnight at 4 °C before transfer to 30% sucrose for cryoprotection for several days. Cryoprotected brain samples were embedded in Tissue-Tek OCT compound (Sakura, 4583) and sectioned in the coronal plane at 50 µm using a sliding microtome (Microm HM450, Thermo Fisher Scientific). TUNEL assays were performed using the Click-iT Plus TUNEL Assay Kit from Thermo Fisher Scientific (C10617). For immunohistochemistry, selected coronal sections were rinsed three times in TBS and incubated in blocking solution (3% normal donkey serum, Jackson Immuno Research, AB2337258; 0.3% Triton X-100 in TBS) at room temperature for 30 minutes. Rabbit anti-Olig2 (1:400, Millipore, AB9610), goat anti-PDGFRα (1:200, R&D Systems, AF1062), mouse anti-APC (CC1, 1:50, Calbiochem, OP80) and rat anti-Ki67 (1:200, Life Technologies, 14-5698-82) were diluted in staining solution (1% normal donkey serum, 0.3% Triton X-100 in TBS) and incubated with sections at room temperature for 2.5 days; when staining for CC1, sections were incubated at 4 °C for 10 days. In other studies, sections were incubated with rabbit anti-SOX9 (1:500, Abcam, AB185966), mouse anti-GFAP (1:200, Thermo Fisher Scientific, 14-9892-82), rabbit anti-Iba1 (1:1,000, Wako, 019-19741) and/or rat anti-CD68 (1:200, Abcam, AB53444) overnight at 4 °C. Sections were then rinsed three times in TBS and incubated in secondary antibody solution containing Alexa Fluor 488 donkey anti-rabbit IgG (1:500, Jackson Immuno Research, 711-545-152), Alexa Fluor 647 donkey anti-goat IgG (1:500, Jackson Immuno Research, A21447), Alexa Fluor 594 donkey anti-mouse IgG (1:500, Jackson ImmunoResearch, 715-585-150), Alexa Fluor 647 donkey anti-mouse IgG (1:500, Jackson Immuno Research, 715-605-150), Alexa Fluor 594 donkey anti-rat IgG (1:500, Jackson Immuno Research, 712-585-153) and/or Hoechst 33342 (1:2,000, Life Technologies, H3570) in staining solution at 4 °C for 12 hours or at room temperature for 2 hours. Sections were rinsed three times in TBS and mounted with ProLong Gold Mounting Medium (Life Technologies, P36930).

### Confocal microscopy

Representative images shown in Figs. 1 and 3 were taken on a Zeiss LSM800 scanning confocal microscope at ×63 magnification, using ZEN software (version 2.1). Z-stack images for counting microglia, astrocytes and TUNEL^+^ cells were taken on a Zeiss LSM800 scanning confocal microscope at ×20, and CD68^+^ microglia were imaged at ×40 magnification. For measurement of glial fibrillary acidic protein (GFAP) area, images were taken in the corpus callosum using a Zeiss LSM700 scanning confocal microscope at ×40 magnification, using ZEN software (version 2.3). Fluorescent signal was enhanced uniformly in the representative merged images to enable visualization of all fluorophores and their co-localization.

### Electron microscopy

Rats were given a lethal dose of Fatal Plus and transcardially perfused with 40–80 ml of ice-cold 0.1 M PBS, followed by 40–80 ml of Karnovsky fixative, consisting of 4% PFA (Electron Microscopy Services (EMS), 15700) and 2% glutaraldehyde (EMS, 16000) in 0.1 M sodium cacodylate buffer (EMS, 12300)^7,11^ in PBS before brain extraction and tissue processing. Mice were given a lethal dose of Fatal Plus or Avertin and transcardially perfused with 10–20 ml of ice-cold PBS followed by 10–20 ml of Karnovsky fixative. Samples were post-fixed in Karnovsky fixative for at least 2 weeks. A ~1-mm^3^ block of tissue was dissected from the midline sagittal corpus callosum body, at the rostrocaudal location overlying the dentate gyrus and the hippocampal fornix, enabling cross-sectional views of callosal projection axons. Another ~1mm^3^ block was taken from the corpus callosum splenium. The dissected block was processed for transmission electron microscopy as described previously^4^. In brief, tissue was post-fixed in 1% osmium tetroxide (EMS, 19100) for 1 hour at room temperature, washed three times in ultra-filtered water, en bloc stained for 2 hours at room temperature before dehydration in gradient ethanols and then rinsed in 100% ethanol twice, followed by acetonitrile (Thermo Fisher Scientific, A21-1). Samples were then embedded in 1:1 EMbed-812 (EMS, 14120):acetonitrile, followed by EMbed-812 for 2 hours and then placed into TAAB capsules filled with fresh resin before incubation in a 65 °C oven overnight. Next, 75–90-nm sections from this block were mounted on formvar/carbon-coated slot grids and contrast stained for 30 seconds in 3.5% uranyl acetate in 50% acetone (EMS, 10015), followed by 0.2% lead citrate (EMS, 0378) for 30 seconds. Samples were imaged with a JEOL JEM-1400 transmission electron microscope at 120 kV, and images were collected with a Gatan Orius digital camera.

### Quantification of TUNEL, microglia and astrocytes

Fiji software (https://imagej.net/software/fiji/, version 2.1.0) was used to quantify cell number per callosal volume (TUNEL, microglia and astrocyte counts). ImageJ software (version 2.0) was used to quantify the area occupied by GFAP in ×40 images from the corpus callosum. For each mouse, 177–379 Iba1^+^ microglia were counted from six fields from corpus callosum taken in three sections, and 577–906 astrocytes were counted from six fields in corpus callosum, taken in three separate brain sections. % GFAP area per field: for each mouse, 12 fields from corpus callosum were quantified.

### Unbiased stereology

OPCs and oligodendrocytes were visualized with an MBF Zeiss Axiocam light microscope. Cell numbers were determined through unbiased stereology using Stereo Investigator software (MBF Bioscience, versions 2017–2019). Regions of interest containing the body of the corpus callosum, beginning rostrally at the level of the hippocampal fornix at the third ventricle and continuing caudally through the anterior hippocampus (approximate bregma AP −0.8 mm to −3.8 mm for rats, −0.8 mm to −2 mm for mice), were traced within sections at ×2.5 magnification. Images were acquired at ×40 from every 6th section throughout this region. For mice, this included 3–6 sections per animal, whereas, for rats, this included 4–7 sections per animal; there were no differences in the number of sections used between experimental groups. Stereological parameters were determined through pilot studies, ensuring that at least 100–300 cells would be counted per animal and the Gunderson m = 1 coefficient of error (CE) was less than 0.1^51,52^ (typical CEs were 0.03–0.07). Exposure time was kept uniform across all samples imaged within each experiment. In all studies, disector height of 30 mm and guard zones of 3 mm were applied unless otherwise indicated. We measured the volume of the body of the corpus callosum (region interrogated for cell counts) with the Cavalieri method^53^. The following stereological parameters were used:

- Mouse OPCs: sampling grid size was set to 225 × 225 μm; counting frame was set to 100 × 100 μm.
- Mouse oligodendrocytes: sampling grid: 500 × 250 μm; counting frame: 100 × 100 μm; disector height: 20 μm. In studies in which TSA or vehicle was administered (Fig. 5), sampling was increased given the use of hemi-brains: sampling grid: 225 × 225 μm; counting frame: 100 × 100 μm; disector height: 24 μm.
- Rat OPCs: 300 × 175 μm; counting frame: 130 × 130 μm.
- Rat oligodendrocytes: 750 × 350 μm; counting frame: 75 × 75 μm; disector height: 20 μm.

Rat OPC counts: At the 1.5-month timepoint, 426–734 cells were counted per rat, whereas 211–448 cells were counted per rat at the 6-month timepoint. Rat oligodendrocyte counts: At the 1.5-month timepoint, 478–1,102 cells were counted for each rat, whereas, at the 6-month timepoint, 703–1,522 cells were counted for each rat.

Mouse OPC counts: For each P21 mouse, 220–316 Ki67^+^ OPCs were counted; for each P45 mouse, 174–347 Ki67^+^ OPCs were counted (Fig. 3). Mouse oligodendrocyte counts: For each P21 mouse, 380–748 mature oligodendrocytes were counted; for each P45 mouse, 555–2,226 mature oligodendrocytes were counted (Fig. 3). TSA treatment studies: for each mouse, 2,256–3,529 oligodendrocytes were counted (Fig. 5).

### Quantitative analysis of myelin structure

We quantified the g-ratio, defined as the axonal diameter in its short axis divided by the diameter of the entire fiber in the same axis (axonal diameter / axonal diameter + myelin sheath), using ImageJ software (https://imagej.nih.gov/ij/)^4,7,35,54^. Myelinated and unmyelinated axons were quantified from ×4,000 transmission electron micrographs. The percentage of myelinated axons was calculated as 100 × (myelinated / (myelinated + unmyelinated) axons). Total (myelinated + unmyelinated) axon number was determined by quantifying the average number of axons per ×4,000 electron micrograph.

Rat g-ratios: In experiments comparing myelination in 1.5- and 6-month-old rats, for each rat, 195–264 axons were measured from 8–18 electron micrographs (Fig. 1). In studies involving ethosuximide administration, for each rat, 184–284 axons from 8–15 electron micrographs were quantified (Fig. 2). In studies of myelination in the corpus callosum splenium, for each rat, 197–217 axons from 10–16 electron micrographs were quantified (Extended Data Fig. 5).

Mouse g-ratios and percent myelination: In experiments comparing g-ratios between P21 and P45 mice: for each P21 mouse, 171-271 axons were quantified from 20-27 electron micrographs. For each P45 mouse, 214-298 axons were measured from 16-20 electron micrographs (Fig. 3). Measurement of percent myelination: for each P21 mouse, 1,311–2,415 unmyelinated axons and 130–227 myelinated axons were counted from a total of ten electron micrographs. For each P45 mouse, 573–1,791 unmyelinated axons and 182–310 myelinated axons were counted from a total of ten electron micrographs. In experiments involving *Scn8a*^+/mut^ cKO mice, for each mouse, 186–218 axons were quantified from 8–14 fields (Fig. 4).

### EEG analysis

EEG data acquired with Open Ephys software were displayed, and seizures from 3–4 hours of EEG recording were visually identified, marked and tabulated by a blinded reviewer using custom MATLAB software, version R2019b. We have found that 3–4 hours of EEG recording is sufficient for reliable measurement of seizure frequency in *Scn8a*^+/mut^ mice^30^ and Wag/Rij rats, similarly to other investigators^29,55^. Seizures were associated with behavioral arrest, and the corresponding EEG demonstrated predominantly 4–8-Hz frequency spike-wave morphology, amplitude ~1.5–2 times that of the background and duration of more than 1 second^30,50^. Coherence was calculated using an adapted version of MATLAB coherence software (https://www.mathworks.com/help/wavelet/ref/wcoherence.html). The custom software used for quantitative analysis of EEG is available on GitHub: https://github.com/huguenardlab/EEG.

### Statistical analysis

All data collection and analysis were performed by experimenters blinded to animal identity and experimental condition. Full details of statistical analyses can be found in the figure legends. For all studies, *n* refers to the number of mice or rats included in each experimental group, and, unless indicated otherwise (for example, g-ratio scatter plots), each data point in a graph represents the mean from one mouse or one rat. For all studies, *n* = 3 or more mice or rats per group, with the exact *n* specified in the figure legends. Sample sizes were based on the variance of data in pilot experiments and were generally estimated by power calculations that determined the number of animals needed for 80% power to detect a 20–30% difference between genotypes. GraphPad Prism software (versions 8 and 9) was used to perform statistical analyses. Statistical significance was defined as *P* < 0.05 throughout. Rarely, data were excluded for the following pre-established reasons: (1) samples incurred damage during histological processing that precluded accurate analysis; (2) EEG recordings were not of sufficient quality to enable accurate interpretation (for example, one channel not working or presence of a major artifact); and (3) statistical outliers, defined as data points >2 standard deviations from the group mean. The Shapiro–Wilk test was used to determine whether data were normally distributed; parametric tests were used where indicated. Non-parametric tests were used for non-parametric datasets. One-way ANOVA followed by Sidak’s post hoc test, correcting for multiple comparisons, was used to compare two groups at specific ages (for example, Figs. 1 and 3 and Extended Data Figs. 1a,b, 2a,c, 3, 6a,e and 9). When two experimental groups within only one time point were compared (Fig. 4i and Extended Data Figs. 1c, 5c, 6b–d, 7 and 10), a two-tailed *t*-test was used. For experiments in which rats were treated with ETX (Fig. 2 and Extended Data Fig. 2b); mice were treated with TSA (Fig. 5, and Extended Data Fig. 9b); and for those studies involving conditional knockout of TrkB from OPCs in *Scn8a*^+/+^ and *Scn8a*^+/mut^ mice (Fig. 4 and Extended Data Fig. 2d), ANOVA with post hoc Tukey’s test was employed to perform multiple comparisons with appropriate corrections. For comparisons of hourly seizure frequency in rats (Fig. 2b), the non-parametric Kruskal–Wallis and Dunn’s post hoc tests were used. For experiments in which rats were treated with ETX, or in which mice were treated with TSA, rats or mice within each litter were randomized to treatment groups.

### Reporting Summary

Further information on research design is available in the Nature Research Reporting Summary linked to this article.

## Online content

Any methods, additional references, Nature Research reporting summaries, source data, extended data, supplementary information, acknowledgements, peer review information; details of author contributions and competing interests; and statements of data and code availability are available at 10.1038/s41593-022-01052-2.

## Supplementary information

Supplementary Information Supplementary Notes 1 and 2.

Reporting Summary

## Data Availability

Raw data are available in the source data files for Figs. 1–5 and Extended Data Figs. 1–10. Source data are provided with this paper.

## Source data

Source Data Fig. 1 Raw Source Data
Source Data Fig. 2 Raw Source Data
Source Data Fig. 3 Raw Source Data
Source Data Fig. 4 Raw Source Data
Source Data Fig. 5 Raw Source Data
Source Data Extended Data Fig. 1 Raw Source Data
Source Data Extended Data Fig. 2 Raw Source Data
Source Data Extended Data Fig. 3 Raw Source Data
Source Data Extended Data Fig. 4 Raw Source Data
Source Data Extended Data Fig. 5 Raw Source Data
Source Data Extended Data Fig. 6 Raw Source Data
Source Data Extended Data Fig. 7 Raw Source Data
Source Data Extended Data Fig. 8 Raw Source Data
Source Data Extended Data Fig. 9 Raw Source Data
Source Data Extended Data Fig. 10 Raw Source Data

## Code availability

Custom MATLAB code used for EEG analyses is available at https://github.com/huguenardlab/EEG.

## References

[1] Mensch S (2015). Synaptic vesicle release regulates myelin sheath number of individual oligodendrocytes in vivo. Nat. Neurosci..

[2] Hines JH, Ravanelli AM, Schwindt R, Scott EK, Appel B (2015). Neuronal activity biases axon selection for myelination in vivo. Nat. Neurosci..

[3] Makinodan M, Rosen KM, Ito S, Corfas G (2012). A critical period for social experience-dependent oligodendrocyte maturation and myelination. Science.

[4] Gibson EM (2014). Neuronal activity promotes oligodendrogenesis and adaptive myelination in the mammalian brain. Science.

[5] Hughes EG, Orthmann-Murphy JL, Langseth AJ, Bergles DE (2018). Myelin remodeling through experience-dependent oligodendrogenesis in the adult somatosensory cortex. Nat. Neurosci..

[6] Mitew S (2018). Pharmacogenetic stimulation of neuronal activity increases myelination in an axon-specific manner. Nat. Commun..

[7] Steadman PE (2020). Disruption of oligodendrogenesis impairs memory consolidation in adult mice. Neuron.

[8] Liu J (2012). Impaired adult myelination in the prefrontal cortex of socially isolated mice. Nat. Neurosci..

[9] Swire, M., Kotelevtsev, Y., Webb, D. J., Lyons, D. A. & Ffrench-Constant, C. Endothelin signalling mediates experience-dependent myelination in the CNS. *eLlife* **8**, e49493 (2019). 10.7554/eLife.49493 PMC6831104 31657718

[10] Yang, S. M., Michel, K., Jokhi, V., Nedivi, E. & Arlotta, P. Neuron class-specific responses govern adaptive myelin remodeling in the neocortex. *Science* **370**, eabd2109 (2020). 10.1126/science.abd2109 PMC7931669 33335032

[11] Geraghty AC (2019). Loss of adaptive myelination contributes to methotrexate chemotherapy-related cognitive impairment. Neuron.

[12] Pajevic S, Basser PJ, Fields RD (2014). Role of myelin plasticity in oscillations and synchrony of neuronal activity. Neuroscience.

[13] Noori R (2020). Activity-dependent myelination: a glial mechanism of oscillatory self-organization in large-scale brain networks. Proc. Natl Acad. Sci. USA.

[14] Kato D (2020). Motor learning requires myelination to reduce asynchrony and spontaneity in neural activity. Glia.

[15] McKenzie IA (2014). Motor skill learning requires active central myelination. Science.

[16] Xiao L (2016). Rapid production of new oligodendrocytes is required in the earliest stages of motor-skill learning. Nat. Neurosci..

[17] Pan S, Mayoral SR, Choi HS, Chan JR, Kheirbek MA (2020). Preservation of a remote fear memory requires new myelin formation. Nat. Neurosci..

[18] Chahboune H (2009). DTI abnormalities in anterior corpus callosum of rats with spike-wave epilepsy. Neuroimage.

[19] Gross DW (2011). Diffusion tensor imaging in temporal lobe epilepsy. Epilepsia.

[20] Yang T (2012). White matter impairment in the basal ganglia-thalamocortical circuit of drug-naive childhood absence epilepsy. Epilepsy Res..

[21] Hatton SN (2020). White matter abnormalities across different epilepsy syndromes in adults: an ENIGMA-Epilepsy study. Brain.

[22] Sandoval Karamian AG, Wusthoff CJ, Boothroyd D, Yeom KW, Knowles JK (2020). Neonatal genetic epilepsies display convergent white matter microstructural abnormalities. Epilepsia.

[23] Goldsberry G, Mitra D, MacDonald D, Patay Z (2011). Accelerated myelination with motor system involvement in a neonate with immediate postnatal onset of seizures and hemimegalencephaly. Epilepsy Behav..

[24] Duprez T (1998). Focal seizure-induced premature myelination: speculation from serial MRI. Neuroradiology.

[25] Guerrini R, Marini C, Barba C (2019). Generalized epilepsies. Handb. Clin. Neurol..

[26] Niedermeyer, E. *Electroencephalography: Basic Principles, Clinical Applications, and Related Fields* (Urban & Schwarzenberg, 2000).

[27] Fogerson PM, Huguenard JR (2016). Tapping the brakes: cellular and synaptic mechanisms that regulate thalamic oscillations. Neuron.

[28] Musgrave J, Gloor P (1980). The role of the corpus callosum in bilateral interhemispheric synchrony of spike and wave discharge in feline generalized penicillin epilepsy. Epilepsia.

[29] Blumenfeld H (2008). Early treatment suppresses the development of spike-wave epilepsy in a rat model. Epilepsia.

[30] Makinson CD (2017). Regulation of thalamic and cortical network synchrony by Scn8a. Neuron.

[31] Brigo F (2018). A brief history of typical absence seizures—petit mal revisited. Epilepsy Behav..

[32] Huntsman MM, Porcello DM, Homanics GE, DeLorey TM, Huguenard JR (1999). Reciprocal inhibitory connections and network synchrony in the mammalian thalamus. Science.

[33] Meeren HK, Pijn JP, VanLuijtelaar EL, Coenen AM, Lopes da Silva FH (2002). Cortical focus drives widespread corticothalamic networks during spontaneous absence seizures in rats. J. Neurosci..

[34] McCafferty C (2018). Cortical drive and thalamic feed-forward inhibition control thalamic output synchrony during absence seizures. Nat. Neurosci..

[35] Smith RS, Koles ZJ (1970). Myelinated nerve fibers: computed effect of myelin thickness on conduction velocity. Am. J. Physiol..

[36] Papale LA (2009). Heterozygous mutations of the voltage-gated sodium channel *SCN8A* are associated with spike-wave discharges and absence epilepsy in mice. Hum. Mol. Genet..

[37] Gibson EM (2019). Methotrexate chemotherapy induces persistent tri-glial dysregulation that underlies chemotherapy-related cognitive impairment. Cell.

[38] Mount, C. W., Yalcin, B., Cunliffe-Koehler, K., Sundaresh, S. & Monje, M. Monosynaptic tracing maps brain-wide afferent oligodendrocyte precursor cell connectivity. *eLife* **8**, e49291 (2019). 10.7554/eLife.49291 PMC6800000 31625910

[39] Ferraro TN (1999). Mapping loci for pentylenetetrazol-induced seizure susceptibility in mice. J. Neurosci..

[40] Morse E (2019). Historical trend toward improved long-term outcome in childhood absence epilepsy. Epilepsy Res..

[41] Larson, V. A. et al. Oligodendrocytes control potassium accumulation in white matter and seizure susceptibility. *eLife* **7**, e34829 (2018). 10.7554/eLife.34829 PMC5903864 29596047

[42] Alam, M. M. et al. Deficiency of microglial autophagy increases the density of oligodendrocytes and susceptibility to severe forms of seizures. *eNeuro* **8**, ENEURO.0183-20.2021 (2021). 10.1523/ENEURO.0183-20.2021 PMC7890520 33472865

[43] Sharma P (2017). Differences in white matter structure between seizure prone (FAST) and seizure resistant (SLOW) rat strains. Neurobiol. Dis..

[44] Tomassy GS (2014). Distinct profiles of myelin distribution along single axons of pyramidal neurons in the neocortex. Science.

[45] Battefeld A, Klooster J, Kole MH (2016). Myelinating satellite oligodendrocytes are integrated in a glial syncytium constraining neuronal high-frequency activity. Nat. Commun..

[46] Xin W (2019). Oligodendrocytes support neuronal glutamatergic transmission via expression of glutamine synthetase. Cell Rep..

[47] Funfschilling U (2012). Glycolytic oligodendrocytes maintain myelin and long-term axonal integrity. Nature.

[48] Shi P (2011). Synapse microarray identification of small molecules that enhance synaptogenesis. Nat. Commun..

[49] Citraro R (2020). Effects of histone deacetylase inhibitors on the development of epilepsy and psychiatric comorbidity in WAG/Rij Rats. Mol. Neurobiol..

[50] Sorokin JM (2017). Bidirectional control of generalized epilepsy networks via rapid real-time switching of firing mode. Neuron.

[51] Slomianka L, West MJ (2005). Estimators of the precision of stereological estimates: an example based on the CA1 pyramidal cell layer of rats. Neuroscience.

[52] West MJ (2013). Getting started in stereology. Cold Spring Harb. Protoc..

[53] West MJ (2012). Estimating volume in biological structures. Cold Spring Harb. Protoc..

[54] Waxman SG (1980). Determinants of conduction velocity in myelinated nerve fibers. Muscle Nerve.

[55] Karson A (2021). Etanercept rescues cognitive deficits, depression-like symptoms, and spike-wave discharge incidence in WAG/Rij rat model of absence epilepsy. Epilepsy Behav..

