# Supplementary material for: Maladaptive myelination promotes generalized epilepsy progression
Source: Nat Neurosci. 2022 May 2;25(5):596–606. doi: 10.1038/s41593-022-01052-2 (PMC9076538; doi:10.1038/s41593-022-01052-2)
Supplement: Supplementary file 1 — Supplementary Notes 1 and 2. [file 41593_2022_1052_MOESM1_ESM.pdf]

nature portfolio

https://doi.org/10.1038/s41593-022-01052-2

Supplementary information

Maladaptive myelination promotes generalized epilepsy progression

In the format provided by the authors and unedited

Supplementary Notes

Supplementary Note 1

The role of BDNF signaling in the context of epilepsy is complex. BDNF- and/or TrkB- mediated signaling has been implicated as a contributing factor in epileptogenesis, particularly in models of temporal lobe epilepsy[1-3]. However, augmenting TrkB signaling can reduce *in vitro* epileptiform activity in a cerebral undercut model of traumatic brain injury[4], or in temporal lobe epilepsy models when BDNF is administered after epileptogenesis has already occurred[5]. These apparently divergent roles may be due to cell type specific responses[6], differences in the timing and location of BDNF signaling, and differential activation of distinct BDNF-related canonical or non-canonical JAK/STAT signaling pathways[7,8]. Shifts to pro-BDNF rather than BDNF signaling can promote epileptogenesis[9].

Additional molecular pathways that link neurons, oligodendrogenesis and myelination include the vasoactive peptide endothelin[10]; purinergic signaling[11,12], and neuregulin signaling through oligodendrocyte ErbB receptors[13]. Relevant to epilepsy, which involves hyperexcitable neuronal networks, OPCs express a variety of voltage- and ligand-gated ion channels, some of which are expressed on neuron-OPC synapses, such as AMPA and NMDA receptors that permit calcium signaling[14,15]

Supplementary Note 2

Brain imaging in numerous forms of human epilepsy, including generalized epilepsy, suggests altered white matter structure[16-22], though definitive conclusions cannot be drawn in the absence of gold standard histology (such as electron microscopy). While such imaging studies are often performed in patients with established epilepsy, our findings pertain to relatively early time-points in absence seizure onset and progression and suggest that myelin plasticity may play an early role in determining seizure frequency and/or severity.

Indeed, *loss* of myelin has been reported in some forms of focal epilepsy, where impaired oligodendrogenesis and loss of or decrease in myelination may be related to specific genetic etiologies, blood-brain barrier compromise and auto-inflammation[23,24 25-27]. In these contexts, it has been hypothesized that impaired glial potassium buffering may promote excessive neuronal excitability[24,28,29]. The possibility that different forms of epilepsy might be associated with distinct myelin phenotypes is supported by an array of imaging findings that may vary based on age, epilepsy duration and other factors [16-22].

Notably, our histological analyses focused on callosal projections connecting somatosensory cortices, while the thalamocortical network as a whole comprises larger territories in the brain. Myelination and oligodendrogenesis might vary depending on the brain region examined and its proximity to the seizure onset. Thus, future work should more fully elucidate network level and longitudinal white matter structural change associated with generalized epilepsy.

Another important consideration is that Both Wag/Rij rats and *Scn8a*$^{+/mut}$ mice have seizures that progress and persist indefinitely, whereas seizures spontaneously remit in some human forms of generalized epilepsy such as Childhood Absence Epilepsy[30-32]. The onset and remission of seizures in Childhood Absence Epilepsy is thought to be due to genetically- and developmentally-determined changes affecting neuronal function within the thalamocortical network[33-36]. Given the primacy of these mechanisms, which are likely independent of activity-regulated myelination, the presence of aberrant myelination would not be required for developmental seizure onset, nor would aberrant patterns of myelination preclude seizure remission.

Finally, it is unknown whether maladaptive myelin changes are reversible. Myelin thinning has been observed in the context of sensory deprivation[13,37,38] suggesting that both increases and decreases in myelin may be regulated by changes in activity. During development, microglia

phagocytose myelin sheaths, suggesting a possible mechanism for eliminating excessive or unneeded myelination[39], although the mechanisms of possible activity-regulated decreases in myelin are not fully understood in the adult brain. Given that myelin plasticity can result from neuronal activity, it is possible that epilepsy therapies involving neurostimulation, which modulate neuronal patterns of activity to achieve seizure reduction and cognitive improvement[40,41] also modulate patterns of myelination, though this has not yet been investigated.

1. Dinocourt, C., Gallagher, S.E. & Thompson, S.M. Injury-induced axonal sprouting in the hippocampus is initiated by activation of trkB receptors. *Eur J Neurosci* **24**, 1857-1866 (2006).
2. Gu, B., *et al.* A Peptide Uncoupling BDNF Receptor TrkB from Phospholipase Cgamma1 Prevents Epilepsy Induced by Status Epilepticus. *Neuron* **88**, 484-491 (2015).
3. Heinrich, C., *et al.* Increase in BDNF-mediated TrkB signaling promotes epileptogenesis in a mouse model of mesial temporal lobe epilepsy. *Neurobiol Dis* **42**, 35-47 (2011).
4. Gu, F., Parada, I., Yang, T., Longo, F.M. & Prince, D.A. Partial TrkB receptor activation suppresses cortical epileptogenesis through actions on parvalbumin interneurons. *Neurobiol Dis* **113**, 45-58 (2018).
5. Falcicchia, C., *et al.* Seizure-Suppressant and Neuroprotective Effects of Encapsulated BDNF-Producing Cells in a Rat Model of Temporal Lobe Epilepsy. *Mol Ther Methods Clin Dev* **9**, 211-224 (2018).
6. Gu, F., *et al.* Structural alterations in fast-spiking GABAergic interneurons in a model of posttraumatic neocortical epileptogenesis. *Neurobiol Dis* **108**, 100-114 (2017).
7. Lund, I.V., *et al.* BDNF selectively regulates GABAA receptor transcription by activation of the JAK/STAT pathway. *Sci Signal* **1**, ra9 (2008).
8. Huang, Y.Z., He, X.P., Krishnamurthy, K. & McNamara, J.O. TrkB-Shc Signaling Protects against Hippocampal Injury Following Status Epilepticus. *J Neurosci* **39**, 4624-4630 (2019).
9. Thomas, A.X., *et al.* Rapid Increases in proBDNF after Pilocarpine-Induced Status Epilepticus in Mice Are Associated with Reduced proBDNF Cleavage Machinery. *eNeuro* **3**(2016).
10. Swire, M., Kotelevtsev, Y., Webb, D.J., Lyons, D.A. & Ffrench-Constant, C. Endothelin signalling mediates experience-dependent myelination in the CNS. *Elife* **8**(2019).
11. Welsh, T.G. & Kucenas, S. Purinergic signaling in oligodendrocyte development and function. *J Neurochem* **145**, 6-18 (2018).
12. Stevens, B., Porta, S., Haak, L.L., Gallo, V. & Fields, R.D. Adenosine: a neuron-glial transmitter promoting myelination in the CNS in response to action potentials. *Neuron* **36**, 855-868 (2002).
13. Makinodan, M., Rosen, K.M., Ito, S. & Corfas, G. A critical period for social experience-dependent oligodendrocyte maturation and myelination. *Science* **337**, 1357-1360 (2012).
14. Thornton, M.A. & Hughes, E.G. Neuron-oligodendroglia interactions: Activity-dependent regulation of cellular signaling. *Neurosci Lett* **727**, 134916 (2020).
15. Larson, V.A., Zhang, Y. & Bergles, D.E. Electrophysiological properties of NG2(+) cells: Matching physiological studies with gene expression profiles. *Brain Res* **1638**, 138-160 (2016).
16. Hatton, S.N., *et al.* White matter abnormalities across different epilepsy syndromes in adults: an ENIGMA-Epilepsy study. *Brain* **143**, 2454-2473 (2020).
17. Ciumas, C., *et al.* White matter development in children with benign childhood epilepsy with centro-temporal spikes. *Brain* **137**, 1095-1106 (2014).
18. Gross, D.W. Diffusion tensor imaging in temporal lobe epilepsy. *Epilepsia* **52 Suppl 4**, 32-34 (2011).

19. Nilsson, D., *et al.* Bilateral diffusion tensor abnormalities of temporal lobe and cingulate gyrus white matter in children with temporal lobe epilepsy. *Epilepsy Res* **81**, 128-135 (2008).
20. Sandoval Karamian, A.G., Wusthoff, C.J., Boothroyd, D., Yeom, K.W. & Knowles, J.K. Neonatal genetic epilepsies display convergent white matter microstructural abnormalities. *Epilepsia* **61**, e192-e197 (2020).
21. Yang, T., *et al.* White matter impairment in the basal ganglia-thalamocortical circuit of drug-naive childhood absence epilepsy. *Epilepsy Res* **99**, 267-273 (2012).
22. Goldsberry, G., Mitra, D., MacDonald, D. & Patay, Z. Accelerated myelination with motor system involvement in a neonate with immediate postnatal onset of seizures and hemimegalencephaly. *Epilepsy Behav* **22**, 391-394 (2011).
23. You, Y., *et al.* Myelin damage of hippocampus and cerebral cortex in rat pentylenetetrazol model. *Brain Res* **1381**, 208-216 (2011).
24. de Curtis, M., Garbelli, R. & Uva, L. A hypothesis for the role of axon demyelination in seizure generation. *Epilepsia* **62**, 583-595 (2021).
25. Zucca, I., *et al.* Type II focal cortical dysplasia: Ex vivo 7T magnetic resonance imaging abnormalities and histopathological comparisons. *Ann Neurol* **79**, 42-58 (2016).
26. Ercan, E., *et al.* Neuronal CTGF/CCN2 negatively regulates myelination in a mouse model of tuberous sclerosis complex. *J Exp Med* **214**, 681-697 (2017).
27. Muhlebner, A., *et al.* Myelin Pathology Beyond White Matter in Tuberous Sclerosis Complex (TSC) Cortical Tubers. *J Neuropathol Exp Neurol* **79**, 1054-1064 (2020).
28. Larson, V.A., *et al.* Oligodendrocytes control potassium accumulation in white matter and seizure susceptibility. *Elife* **7**(2018).
29. Battefeld, A., Klooster, J. & Kole, M.H. Myelinating satellite oligodendrocytes are integrated in a glial syncytium constraining neuronal high-frequency activity. *Nat Commun* **7**, 11298 (2016).
30. Blumenfeld, H., *et al.* Early treatment suppresses the development of spike-wave epilepsy in a rat model. *Epilepsia* **49**, 400-409 (2008).
31. Makinson, C.D., *et al.* Regulation of Thalamic and Cortical Network Synchrony by Scn8a. *Neuron* **93**, 1165-1179 e1166 (2017).
32. Wirrell, E.C. Natural history of absence epilepsy in children. *Can J Neurol Sci* **30**, 184-188 (2003).
33. Paolicchi, J.M. The timing of pediatric epilepsy syndromes: what are the developmental triggers? *Ann N Y Acad Sci* **1304**, 45-51 (2013).
34. Heron, S.E., *et al.* Extended spectrum of idiopathic generalized epilepsies associated with CACNA1H functional variants. *Ann Neurol* **62**, 560-568 (2007).
35. Chen, Y., *et al.* Association between genetic variation of CACNA1H and childhood absence epilepsy. *Ann Neurol* **54**, 239-243 (2003).
36. Urak, L., Feucht, M., Fathi, N., Hornik, K. & Fuchs, K. A GABRB3 promoter haplotype associated with childhood absence epilepsy impairs transcriptional activity. *Hum Mol Genet* **15**, 2533-2541 (2006).
37. Yang, S.M., Michel, K., Jokhi, V., Nedivi, E. & Arlotta, P. Neuron class-specific responses govern adaptive myelin remodeling in the neocortex. *Science* **370**(2020).
38. Liu, J., *et al.* Impaired adult myelination in the prefrontal cortex of socially isolated mice. *Nat Neurosci* **15**, 1621-1623 (2012).

39. Hughes, A.N. & Appel, B. Microglia phagocytose myelin sheaths to modify developmental myelination. *Nat Neurosci* **23**, 1055-1066 (2020).
40. Cukiert, A., Cukiert, C.M., Burattini, J.A. & Mariani, P.P. Seizure outcome during bilateral, continuous, thalamic centromedian nuclei deep brain stimulation in patients with generalized epilepsy: a prospective, open-label study. *Seizure* **81**, 304-309 (2020).
41. Li, M.C.H. & Cook, M.J. Deep brain stimulation for drug-resistant epilepsy. *Epilepsia* **59**, 273-290 (2018).
