# Supplementary material for: Maladaptive myelination promotes generalized epilepsy progression
Source: Nat Neurosci. 2022 May 2;25(5):596–606. doi: 10.1038/s41593-022-01052-2 (PMC9076538; doi:10.1038/s41593-022-01052-2)
Supplement: Supplementary file 2 — Reporting Summary [file 41593_2022_1052_MOESM2_ESM.pdf]

nature research

Corresponding author(s): Michelle Monje, MD PhD
John Huguenard, PhD
Juliet Knowles, MD PhD

Last updated by author(s): 3/3/2022

Reporting Summary

Nature Research wishes to improve the reproducibility of the work that we publish. This form provides structure for consistency and transparency in reporting. For further information on Nature Research policies, see our Editorial Policies and the Editorial Policy Checklist.

Statistics

For all statistical analyses, confirm that the following items are present in the figure legend, table legend, main text, or Methods section.

n/a	Confirmed	
☐	☒	The exact sample size (*n*) for each experimental group/condition, given as a discrete number and unit of measurement
☐	☒	A statement on whether measurements were taken from distinct samples or whether the same sample was measured repeatedly
☐	☒	The statistical test(s) used AND whether they are one- or two-sided<br>*Only common tests should be described solely by name; describe more complex techniques in the Methods section.*
☒	☐	A description of all covariates tested
☐	☒	A description of any assumptions or corrections, such as tests of normality and adjustment for multiple comparisons
☐	☒	A full description of the statistical parameters including central tendency (e.g. means) or other basic estimates (e.g. regression coefficient) AND variation (e.g. standard deviation) or associated estimates of uncertainty (e.g. confidence intervals)
☐	☒	For null hypothesis testing, the test statistic (e.g. F, t, r) with confidence intervals, effect sizes, degrees of freedom and P value noted<br>*Give P values as exact values whenever suitable.*
☒	☐	For Bayesian analysis, information on the choice of priors and Markov chain Monte Carlo settings
☒	☐	For hierarchical and complex designs, identification of the appropriate level for tests and full reporting of outcomes
☒	☐	Estimates of effect sizes (e.g. Cohen's *d*, Pearson's *r*), indicating how they were calculated

Our web collection on statistics for biologists contains articles on many of the points above.

Software and code

Policy information about availability of computer code

Data collection	Representative images and images used to quantify TUNEL positive OPCs, TUNEL positive oligodendrocytes, microglia and astrocytes were collected with a Zeiss confocal microscope, LSM800 or LSM700 model, using Zen 2.1 or 2.3 software, as indicated in the Methods. Fluorescent images used to quantify cells with unbiased stereology were collected with Stereo Investigator software (MBF Bioscience, versions 2017-2020). Continuous real-time EEG was recorded with Open Ephys software (https://open-ephys.org, version 0.4.4.1).
Data analysis	Cell counts were performed by unbiased stereology using Stereo Investigator software (MBF Bioscience, versions 2017-2020). ImageJ software (https://imagej.nih.gov/ij/, versions 1.53a - 2.0) and Fiji software (imagej.net/software/fiji/, version 2.1.0) were used to quantify g-ratios from transmission electron micrographs and cell counts where indicated in the Methods. Unmyelinated axons and myelinated axons were counted from transmission electron micrographs. Seizures from EEG recordings and EEG coherence were quantified using custom Matlab software, version R2019B, available at: https://github.com/huguenardlab/EEG. GraphPad Prism software (GraphPad Software, versions 8 and 9) was used to perform statistical analyses.

For manuscripts utilizing custom algorithms or software that are central to the research but not yet described in published literature, software must be made available to editors and reviewers. We strongly encourage code deposition in a community repository (e.g. GitHub). See the Nature Research guidelines for submitting code & software for further information.

Data

Policy information about availability of data

All manuscripts must include a data availability statement. This statement should provide the following information, where applicable:

- Accession codes, unique identifiers, or web links for publicly available datasets
- A list of figures that have associated raw data
- A description of any restrictions on data availability

Data Availability: Raw data is available in the source data files for Figures 1-5 and Extended Data Figures 1-10.
Code availability: Custom Matlab code used for EEG analyses is available at: https://github.com/huguenardlab/EEG

Field-specific reporting

Please select the one below that is the best fit for your research. If you are not sure, read the appropriate sections before making your selection.

☒ Life sciences ☐ Behavioural & social sciences ☐ Ecological, evolutionary & environmental sciences

For a reference copy of the document with all sections, see nature.com/documents/nr-reporting-summary-flat.pdf

Life sciences study design

All studies must disclose on these points even when the disclosure is negative.

Sample size	For all studies, n= 3 or more mice / rats per group, with the exact n specified in figure legends. Sample sizes were based on the variance of data in pilot experiments, and were generally estimated by power calculations which determined the number of animals "n" needed for 80% power to detect a 20-30% difference between genotypes, at the p<0.05 significance level.
Data exclusions	Rarely, data were excluded for the following pre-established reasons: (1) samples incurred damage during histological processing that precluded accurate analysis (2) EEG recordings were not of insufficient quality to enable accurate interpretation (e.g. one channel not working or significant artifact) and (3) statistical outliers, defined as data points > 2 standard deviations from the group mean.
Replication	To ensure reproducibility, individual animals utilized came from > or = 2 distinct litters for the majority of experiments, and data were collected in separate, sequential experiments. For all experiments included in this study, data were successfully replicated.
Randomization	In experiments involving drug or vehicle treatment groups, treatment was assigned to individual animals randomly and in a method ensuring equal numbers of male and female animals assigned to each treatment group.
Blinding	All data collection and analyses were performed by experimenters blinded to subject experimental condition.

Reporting for specific materials, systems and methods

We require information from authors about some types of materials, experimental systems and methods used in many studies. Here, indicate whether each material, system or method listed is relevant to your study. If you are not sure if a list item applies to your research, read the appropriate section before selecting a response.

Materials & experimental systems

n/a	Involved in the study	
☐	☒	Antibodies
☒	☐	Eukaryotic cell lines
☒	☐	Palaeontology and archaeology
☐	☒	Animals and other organisms
☒	☐	Human research participants
☒	☐	Clinical data
☒	☐	Dual use research of concern

Methods

n/a	Involved in the study	
☒	☐	ChIP-seq
☒	☐	Flow cytometry
☒	☐	MRI-based neuroimaging

Antibodies

Antibodies used	Primary antibodies: Rabbit anti-Olig2 (1:400, Millipore AB9610, lots 3172075, 2987464 and 3045562), Goat anti-PDGFRα (1:200, R&D Systems AF1062, lots HMQ0218081, HMQ0216021), mouse anti-APC (CC1,1:50, Calbiochem, OP80, lots 3031622, 2834781), or Rat anti-Ki67 (1:200, Life Technologies, 14-5698-82, lots 2056928, 2002315, 4328926), Rabbit anti- SOX9 (1:500, Abcam AB185966. lot GR3241181 - 12), Mouse anti-GFAP (1:200, Thermo Scientific 14-9892-82, lot 2358413), Rabbit anti-Iba1 (1:1000, Wako 019-19741, lot LEK0542), and/or Rat anti-CD68 (1:200, Abcam AB53444, Lot GR3384340-2)<br>Secondary antibodies: Alexa 488 donkey anti-rabbit IgG (1:500, Jackson Immuno Research, 711-545-152, lots 141848, 127725, 127498), Alexa 647 donkey anti-goat IgG (1:500, Jackson Immuno Research, A21447, lot 1977345), Alexa 594 donkey anti-mouse IgG

	(1:500, Jackson Immuno Research, 715-585-150, lots 134514, 124676, 140149), Alexa 594 donkey anti-rat IgG (1:500, Jackson Immuno Research, 712-585-153, lot 134903), or Alexa 647 donkey anti-mouse IgG (1:500, Jackson Immuno Research, 715-605-150, lot 155989);
Validation	Millipore Rabbit anti-Olig2, R&D Systems Goat anti-PDGFRα and Calbiochem mouse anti-APC(CC1) as listed above have each been validated and used in rodent brain tissue in multiple previous publications (e.g. Gibson and Monje, Science 2014 PMID 24727982; Geraghty and Monje, Neuron 2019 PMID 31122677). Throughout the manuscript, representative photomicrographs are shown which clearly resemble those of prior publications (Figures 1, 3; for comparison see publications PMID 31018125, 24727982). In brain tissue from mice and rats used in this study, cells which co-expressed Olig2 and PDGFRa expressed in a characteristic ramified pattern around the nuclear Olig2 stain were considered to be oligodendrocyte precursor cells (similar to Dang et al, Cell Reports 2019, PMID 31018125, shown in Figures 1 and 3). To identify dividing OPCs, we performed immunostaining with Life Technologies Rat anti-Ki67 and counted Ki67-expressing OPCs. This Ki67 antibody has been used to label dividing cells in the mouse brain (for example, see Arimura et al, PMID 30422377) and clearly co-localized with the nuclear stain Olig2 in mice and rats (Figures 1 and 3). Mature oligodendrocytes co-expressed CC1 (peri-nuclear) and Olig2 (nuclear) as previously demonstrated in Gibson and Monje, Science 2014 PMID 24727982. Abcam anti-Sox9 and Thermo Fisher anti-GFAP have been previously used to label astrocytes in vivo in mice, in published work (PMIDs 31433295, 32381088) and cells co-labeled with Sox9 and GFAP exhibited morphology consistent with astrocytes as shown in representative photomicrographs in the manuscript. Wako anti-Iba1 and Abcam Rat anti-CD68 have been used by our laboratory to study microglial reactivity in previously published work: Gibson and Monje, Cell 2019, PMID 30528430.<br><br>In addition, antibodies were validated by the company from which each antibody was purchased:<br>Anti-Olig2: https://www.emdmillipore.com/US/en/product/Anti-Olig-2-Antibody,MM_NF-AB9610; confirmed reactivity in mice and rats<br>Anti-PDGFRa: https://www.rndsystems.com/products/mouse-pdgf-ralpha-antibody_af1062; confirmed reactivity in mice; reactivity in rats confirmed as above<br>Anti-Ki67: https://www.thermofisher.com/antibody/product/Ki-67-Antibody-clone-SolA15-Monoclonal/14-5698-82; confirmed reactivity in mice and rats<br>Anti-CC1: https://www.emdmillipore.com/US/en/product/Anti-APC-Ab-7-Mouse-mAb-CC-1,EMD_BIO-OP80; confirmed reactivity in mice and rats<br>Anti-Sox9: https://www.abcam.com/sox9-antibody-epr14335-78-ab185966.html; confirmed reactivity in mice and rats<br>Anti-GFAP: https://www.thermofisher.com/antibody/product/GFAP-Antibody-clone-GA5-Monoclonal/14-9892-82, confirmed reactivity in mice and rats<br>Anti-Iba1: https://labchem-wako.fujifilm.com/us/product/detail/W01W0101-1974.html, confirmed reactivity in mice and rats<br>Anti-CD68: https://www.abcam.com/cd68-antibody-fa-11-ab53444.html, confirmed reactivity in mice

Animals and other organisms

Policy information about studies involving animals; ARRIVE guidelines recommended for reporting animal research

Laboratory animals	All experiments were conducted in accordance with protocols approved by the Stanford University Institutional Animal Care and Use Committee (IACUC; protocols 27215, 12363 and 33969). Mice or rats were group or single housed (up to 5 mice or 2 rats per cage) according to standard guidelines with ad libitum access to food and water in a 12 hour light/dark cycle. In rooms where mice and rats were housed, the ambient temperature was 70 +/- 2 degrees F and the relative humidity was 30-70%. The strains and ages of all mice and rats used in specific studies are indicated in the figures and throughout the text. Male and female mice were used in all studies. Briefly, in studies using Wag/Rij and control (Wistar) rats, 1.5-month-old rats were used to assess endpoints prior to seizure onset and 6-7-month-old rats were used to assess endpoints after seizures are well established. In studies using Scn8a+/mut mice and wild-type littermates on a C3HeB/FeJ background strain, post-natal day (P)21 mice were used to assess endpoints prior to seizure onset, P28 mice were used to assess timepoints during seizure progression and P45 mice were used to assess endpoints after seizures are well established. In studies in which Scn8a+/mut mice were bred onto a mixed C3HeB/FeJ and C57/BL6 background (Figure 4), because seizure onset is delayed, later time points (3 to 6 months) were used to study seizure progression, as described in detail in the text.
Wild animals	This study did not involve wild animals.
Field-collected samples	This study did not involve samples collected in the field.
Ethics oversight	All experiments were conducted in accordance with protocols approved by the Stanford University Institutional Animal Care and Use Committee (IACUC).

Note that full information on the approval of the study protocol must also be provided in the manuscript.
